# Supplementary material for: Multifractal scaling analyses of urban street network structure: The cases of twelve megacities in China
Source: PLoS One. 2021 Feb 18;16(2):e0246925. doi: 10.1371/journal.pone.0246925 (PMC7891711; doi:10.1371/journal.pone.0246925)
Supplement: S1 Appendix — (DOCX) [file pone.0246925.s001.docx]

**S1 Appendix.** The results of multifractal computation conducted by street nodes.

As a reference, we apply the multifractal computation to the street nodes in the same process. The results are as follows.

### Global multifractal spectrums

Simple fractal dimensions reveal limited but useful information about spatial coverage and connectivity of urban street networks. The basic fractal parameters calculated for different cities are summarized in Table 1. Above all, we focus on three basic fractal dimensions: *capacity dimension* *D*_0_, *information dimension* *D*_1_, and *correlation dimension* *D*_2_. For the most commonly known first-tier cities in China: Beijing city, Shanghai city, and Guangzhou city, their *D*_0_, *D*_1_, *D*_2_ are the largest, implying pretty high space-filling degree and accessibility. This is related to highly developed economic and social activities. Shenzhen city is special: limited by natural reserved areas such as many large ecological parks, the spatial coverage of street nodes is very incomplete (*D*_0_=1.8654); but its connectivity is relatively flexible as *D*_2_ is not low (*D*_2_=1.7041). As for other second-tier cities, such as Tianjin city, Nanjing city, etc., their capacity dimensions are very close to each other (*D*_0_≈1.91). But the differences of spatial connectivity are preliminary showed up by *D*_2_. Among these, we also notice two prominent cities, Shenyang city and Chengdu city, for their higher space- filling degree and accessibility of street networks.

Empirical results illustrate that the spatial pattern of urban street networks bears multifractal structure in Chinese cities. Significantly, *D*_0_>*D*_1_>*D*_2_ holds for all the 12 cities. Fig 1 shows that the generalized correlation dimension *D*(*q*) are all monotonic decreasing curves rather than nearly horizontal lines. In the generalized correlation dimension *D*(*q*) spectrums, the macrostructure of street networks in twelve cities shows both similarities and differences. Although all of them take on similar monotonic decreasing tendency, the *D*(*q*) spectrums of different cities have their respective differences, whether in regards to change rate or range. This means that some cities may have richer or poorer density hierarchies or continuity, which preliminarily reveals that the structural levels of spatial patterns of urban street networks vary greatly in terms of different cities.

**Table 1. Basic fractal parameters of street network for different cities.**

| Region | City | Area  (km^2^) | Capacity dimension | | Information dimension | | Correlation dimension | |
| --- | --- | --- | --- | --- | --- | --- | --- | --- |
|  |  |  | ***D*_0_** | ***R^2^*** | ***D*_1_** | ***R^2^*** | ***D*_2_** | ***R^2^*** |
| North China | Beijing | 2719 | **1.9712***** | *0.9994* | **1.8289***** | *0.9978* | **1.7434***** | *0.9994* |
|  |  |  | (0.0197) |  | (0.0351) |  | (0.0174) |  |
|  | Tianjin | 901 | **1.9114***** | *0.9957* | **1.7592***** | *0.9847* | **1.6603***** | *0.9957* |
|  |  |  | (0.0513) |  | (0.0895) |  | (0.0445) |  |
| South China | Guangzhou | 1660 | **1.9559***** | *0.9988* | **1.8207***** | *0.994* | **1.747***** | *0.9988* |
|  |  |  | (0.0277) |  | (0.0577) |  | (0.0247) |  |
|  | Shenzhen | 1561 | **1.8654***** | *0.9989* | **1.7606***** | *0.9938* | **1.7041***** | *0.9989* |
|  |  |  | (0.0253) |  | (0.0568) |  | (0.0231) |  |
| East China | Shanghai | 2589 | **1.9622***** | *0.9996* | **1.8297***** | *0.9976* | **1.7502***** | *0.9996* |
|  |  |  | (0.0160) |  | (0.0366) |  | (0.0143) |  |
|  | Nanjing | 860 | **1.9165***** | *0.9955* | **1.7733***** | *0.9863* | **1.6776***** | *0.9955* |
|  |  |  | (0.0526) |  | (0.0853) |  | (0.0460) |  |
| Central China | Wuhan | 633 | **1.9101***** | *0.9954* | **1.7851***** | *0.9875* | **1.7084***** | *0.9954* |
|  |  |  | (0.0530) |  | (0.0820) |  | (0.0474) |  |
|  | Zhengzhou | 489 | **1.9059***** | *0.9946* | **1.7606***** | *0.9831* | **1.6641***** | *0.9946* |
|  |  |  | (0.0573) |  | (0.0942) |  | (0.0501) |  |
| West China | Xi’an | 632 | **1.9121***** | *0.9955* | **1.787***** | *0.9844* | **1.7053***** | *0.9955* |
|  |  |  | (0.0525) |  | (0.0918) |  | (0.0468) |  |
|  | Chengdu | 1348 | **1.9367***** | *0.9971* | **1.8096***** | *0.9913* | **1.7254***** | *0.9971* |
|  |  |  | (0.0426) |  | (0.0692) |  | (0.0380) |  |
| Northeast China | Shenyang | 755 | **1.9391***** | *0.997* | **1.834***** | *0.995* | **1.7564***** | *0.997* |
|  |  |  | (0.0434) |  | (0.0531) |  | (0.0393) |  |
|  | Harbin | 326 | **1.8559***** | *0.9923* | **1.702***** | *0.9681* | **1.6091***** | *0.9923* |
|  |  |  | (0.0667) |  | (0.1261) |  | (0.0579) |  |

**Note:** The robust Standard Errors are quoted in parenthesis. ^***^ significant at 1%.

A. B.

**Fig 1. The generalized correlation dimension spectrums of urban street networks for different cities.** The generalized correlation dimension *D*(*q*) curves are all monotonic decreasing functions of *q*, indicating multifractal property. On the one hand, the change rates or ranges of *D*(*q*) vary evidently for different cities. On the other hand, the left tails of *D*(*q*) curve determined by sparse areas of *q*<0, exhibit larger differences, while the right tails of *D*(*q*) curve representing high-density areas of *q>*0, tend to overlap, and the convergence values for some cities are very close.

### Local multifractal spectrums

Local spectrums and parameters bring the local and micro features into focus. Corresponding to the global parameters, the local parameters also display the characteristics of multifractal spectrums. As shown in Fig 2, local singularity spectrums *f*(*α*) are all convex parabolas instead of a single point indicating monofractality. In fact, the local parameter *α*(*q*) is more sensitive than global parameter *D*(*q*). The singularity exponent *α*(*q*) curve displays a similar shape and tendency to *D*(*q*), but it changes more abruptly (Fig 2A). Further multifractal features can be demonstrated as follows.

(A) Singularity exponent, *α*(*q*) (B) Local fractal dimension, *f*(*q*)

(C) Singularity spectrum, *f*(*α*)

**Fig 2. The local multifractal spectrums of different cities.** Take some typical curves in four cities as examples. (A) The singularity exponent *α*(*q*) curve is a monotonic function of the moment order *q*. But the change rate varies in different subareas and in different cities. (B) The local fractal dimension *f*(*q*) curves is a single-peak curve, first increasing then decreasing. (C)The singularity spectrum *f*(*α*) is generally a convex parabola, low on the left and high on the right.

**Firstly, the fractal growth pattern of urban street network presents the characteristics of centripetal agglomeration**. There are two basic models of fractal growth in urban development: one is spatial concentration, whose *f*(*α*) shows a unimodal curve with right deviation; and the other is external expansion, showing left deviation. As can be seen, the values of local dimension *f*(*q*) in the left tail are larger than the right tail (Fig 2B), and ∆*f* <0 (Table 2). On the other hand, local singularity spectrum *α*-*f*(*α*) shows a strongly marked non-symmetric shape, whose left tail is much longer than the right tail, and the spectral density in the right tail is significantly higher (Fig 2C). These phenomena are observed in each city. Together these facts imply that the spatial distribution of urban street nodes is dominated by highly-developed centers, indicating the geographical growth of street network is intensive and central agglomerated.

**Table 2. Multifractal parameters of street nodes for each city by OLS regression method.**

| Region | City | *D_-40_* | *D*_+40_ | *α_-40_* | *α_+40_* | *f*_-40_ | *f*_+40_ | Δ*f* |
| --- | --- | --- | --- | --- | --- | --- | --- | --- |
| North China | Beijing | **2.8813***** | **1.4959***** | **2.9285***** | **1.4623***** | **0.9936**** | **0.15049**** | -1.2838 |
|  |  | (0.3628) | (0.0473) | (0.3842) | (0.0472) | (0.3268) | (0.0490) |  |
|  | Tianjin | **2.6968***** | **1.3558***** | **2.7349***** | **1.3268***** | **1.1759***** | **0.1962**** | -1.4259 |
|  |  | (0.5016) | (0.0543) | (0.5299) | (0.0544) | (0.2879) | (0.0704) |  |
| South China | Guangzhou | **2.929***** | **1.4272***** | **2.976***** | **1.3934***** | **1.047**** | **0.0751** | -1.4907 |
|  |  | (0.4898) | (0.0601) | (0.5191) | (0.0602) | (0.3209) | (0.1857) |  |
|  | Shenzhen | **2.9537***** | **1.4708***** | **3.003***** | **1.4384***** | **0.978**** | **0.1721** | -1.2805 |
|  |  | (0.7577) | (0.0325) | (0.7967) | (0.0343) | (0.2689) | (0.2097) |  |
| East China | Shanghai | **3.0571***** | **1.4621***** | **3.1092***** | **1.4271***** | **0.974**** | **0.0585** | -1.3737 |
|  |  | (0.6649) | (0.0525) | (0.6978) | (0.0521) | (0.3058) | (0.0520) |  |
|  | Nanjing | **2.6646***** | **1.2961***** | **2.7023***** | **1.2647***** | **1.15394**** | **0.0423** | -1.7014 |
|  |  | (0.4891) | (0.0602) | (0.5185) | (0.0597) | (0.3433) | / |  |
| Central China | Wuhan | **2.6171***** | **1.4139***** | **2.654***** | **1.3824***** | **1.1386**** | **0.1541***** | -1.4410 |
|  |  | (0.3903) | (0.0362) | (0.4143) | (0.0355) | (0.3307) | (0.0390) |  |
|  | Zhengzhou | **2.6086***** | **1.3304***** | **2.6451***** | **1.2974***** | **1.1476**** | **0.0109** | -1.6734 |
|  |  | (0.4702) | (0.0199) | (0.4997) | (0.0192) | (0.3418) | / |  |
| West China | Xi’an | **2.6181***** | **1.3591***** | **2.6548***** | **1.3271***** | **1.1509**** | **0.0796** | -1.6156 |
|  |  | (0.4727) | (0.0489) | (0.5018) | (0.0498) | (0.3401) | (0.1819) |  |
|  | Chengdu | **2.6633***** | **1.3584***** | **2.701***** | **1.3261***** | **1.1552**** | **0.0666** | -1.6757 |
|  |  | (0.3730) | (0.0672) | (0.3956) | (0.0668) | (0.3174) | / |  |
| Northeast China | Shenyang | **2.5199***** | **1.4075***** | **2.5551***** | **1.3738***** | **1.1135**** | **0.0575** | -1.5086 |
|  |  | (0.2443) | (0.0445) | (0.2602) | (0.0448) | (0.3444) | (0.1003) |  |
|  | Harbin | **2.6261***** | **1.3225***** | **2.6613***** | **1.2899***** | **1.216***** | **0.0184** | -1.6623 |
|  |  | (0.6637) | (0.0560) | (0.7000) | (0.0545) | (0.2726) | / |  |

**Note:** The robust Standard Errors are quoted in parenthesis. ^***^ significant at 1%; ^**^ significant at 5%; ^*^ significant at 10%.

**Second, the fractal structure of urban street network in high-density areas has been arranged in good order, while that in sparse areas show disorders and chaos.** With the changes of moment order *q*, probability structures are reconstructed by *μ*-weighted method. Different subsets in multifractals are examined, variable extent of structural disorders in urban street networks can be revealed. Generally, the sparse regions show more abnormalities. One marker is that the value of *D*(*q*) when *q*<0 exceeds the Euclidean dimension of the embedding space *d*_E_=2, which is abnormal (Fig 1). It is because when *q*<0 and approaches -40, large amounts of subareas with pretty low density (sparse regions) are greatly magnified, rather the main urban areas are hidden, causing massive overlapping subsets. As a result, fractal dimension would break the embedding dimension 2 sooner or later. According to the results displayed in Table 2, the convergence value *D*_-40_ and *α*_-40_ in Shanghai, Shenzhen and Guangzhou break the limitation more severely, even near 3. This suggests that the dilemma of chaotic structure of street networks may be serious in sparse areas. The other marker of disorder is that the goodness of fit for estimating local parameters *α*(*q*) and *f*(*q*) declined with the decreasing of *q*. That is, if *q*→-∞, the scattered points on the log-log plots for direct determination of *α*(*q*) and *f*(*q*) fall into disarray to different extent. Take *f*(*q*) parameters as examples. As shown in Fig 3, when the value of *q* is changed to -2, -5, -10 and -20, the scatters in log-log plot become more and more chaotic correspondingly. Comparatively, the goodness of fit for Shenzhen is relatively better when it experiences the same degree of distortion by *q*. As *q* decreases, most of scatters still remain stable in the log-log plots, indicating a more stable fractal structure. In contrast, Beijing and Xi’an are more disordered and their fitting effects suffer worse degeneration.

(A) Beijing: *q*=-2 (B) Beijing: *q*=-5 (C) Beijing: *q*=-10 (D) Beijing: *q*=-20

(E) Shenzhen: *q*=-2 (F) Shenzhen: *q*=-5 (G) Shenzhen: *q*=-10 (H) Shenzhen: *q*=-20

(I) Xi’an: *q*=-2 (J) Xi’an: *q*=-5 (K) Xi’an: *q*=-10 (L) Xi’an: *q*=-20

(M) Shenyang: *q*=-2 (N) Shenyang: *q*=-5 (O) Shenyang: *q*=-10 (P) Shenyang: *q*=-20

**Fig 3. The log-log plots for estimating the local fractal dimension *f*(*q*) with changes of moment order *q*.** With the decreasing of *q*, the scattered points in log-log plots become more and more disordered, and the goodness of fit for *f*(*q*) estimation declined to some extent.
