# Supplementary material for: Multifractal scaling analyses of urban street network structure: The cases of twelve megacities in China
Source: PLoS One. 2021 Feb 18;16(2):e0246925. doi: 10.1371/journal.pone.0246925 (PMC7891711; doi:10.1371/journal.pone.0246925)
Supplement: Attachment — Submitted filename: Response to Reviewers.docx [file pone.0246925.s006.docx]

## Responses to Editor and Reviewers

### 1 How to revise this manuscript

We have revised the manuscript, according to the comments and suggestions of reviewers, and respond, point by point to, the comments as listed below. Revised portions are marked in red in the paper. The following summarizes how we responded to reviewer comments.

List of Major Changes:

1) The title and abstract have been revised.
2) We have reorganized the Introduction section to better clarify the objectives and framework of the paper.
3) We have reorganized the Multifractal methodology section to be more understandable. And a paragraph was added to further explain the differences between multifractals and monofractals.
4) A table was added to the text to explain the geographical meanings of three simple fractal dimensions for traffic networks (Table 1).
5) A figure was added to the text to explain the application of multifractal analysis to traffic networks (Fig 2). And the original Fig 1 was removed.
6) Considering the reviewer’s suggestions, we have replaced the street nodes with street links as measurements, and recalculated the multifractal parameters of urban street networks by the conventional ordinary least square regression. The goodness of fit (*R*^2^), the *p*-value, and the standard error of each parameter are given in Table 2, Table 3, and S3 Table. The analysis results and conclusions have been adjusted accordingly.
7) A paragraph was added to illustrate the details of identifying urban boundary by city clustering algorithm (CCA).
8) The original Fig 8 has been removed.
9) The Discussion section and Conclusion section have been rewritten.

### 2 Editor Comments:

**1.**	Please ensure that your manuscript meets PLOS ONE's style requirements, including those for file naming.

**[Reply]** This manuscript has been revised to meet PLOS ONE's style requirements.

**2.**	We suggest you thoroughly copyedit your manuscript for language usage, spelling, and grammar.

**[Reply]** We have revised the manuscript carefully.

**3.**	Please update your data availability statement to detail how the Chinese digital navigation map data can be accessed by future researchers.

**[Reply]** The data support from "Geographic Data Sharing Infrastructure, College of Urban and Environmental Sciences, Peking University (http://geodata.pku.edu.cn)".

**4.**	We note that you have provided funding information that is not currently declared in your Funding Statement. Please remove any funding-related text from the manuscript and let us know how you would like to update your Funding Statement.

**[Reply]** The funding-related text in the Acknowledgments section has been removed.

5.	We note that Figures 3, 4 and B1 in your submission contain map images which may be copyrighted.

**[Reply]** The original Figures 3 and 4 were drawn by ourselves by using ArcGIS 10.2. I don’t think they are previously copyrighted maps? And Figure B1 has been removed now.

6.	Please ensure that you refer to Figure 2 in your text as, if accepted, production will need this reference to link the reader to the figure.

**[Reply]** In this revision, we have carefully checked the figures and tables. And the original Figure 2 (now Fig 1) has been linked to Line 149.

### 2.1 Reviewer #1:

The authors propose an interesting method to investigate the multifractal nature of traffic networks and they support their analysis with the example of the 12 most representative cities in China. They show the multi-dimensionality of the fractal space in contrast with the monofractal scale of urban network, when we consider the street nodes as element of the network (preferred respect to the link lines). They highlight the similarities and the divergence among the dataset of 12 cities, trying to explain the causes and the main characteristics.

In my opinion, the results and the methods proposed deserve certainly to be published and the choice of PLOS ONE seems to me the most accurate and appropriate for the journal.

Even if the English used in the paper is correct and in some part (last) also enjoyable, I found an important lack of coherence and order in the formulas and definitions, especially in all the first half of the paper. I warmly suggest to review this part and improve the readability and the clearness.

**1.**	The abstract results too technical and obscure, without proper definition of the cited parameters. It is fine to cite some statistical features but with a minimum of mathematical coherence.

**[Reply]** Thanks for your helpful suggestions. In this revision, the abstract has been revised for better readability. Please refer to Line 10-30:

“Multifractals are self-similar hierarchies with cascade structure, which can be associated with complex networks. Urban traffic networks have been proved to be complex systems with fractal structure. However, previous studies mainly focused on monofractal networks. This paper is devoted to exploring the multifractal scaling in the street network of 12 Chinese cities. The city clustering algorithm (CCA) is employed to identify urban boundaries and define street systems in a consistent manner. Then, box-counting method and the direct determination method based on *μ*-weight are utilized to estimate global and local multifractal parameters. The basic algorithm of parameter estimation is the ordinary least squares method. The results displayed multifractal structure of urban street networks. The global multifractal dimension spectrum is an inverse S-shaped curve, while the local singularity spectrum is an asymmetric unimodal curve. If the moment order *q* approaches negative infinity, the generalized correlation dimension will seriously exceed the embedding space dimension 2, and the local fractal dimension curve displays an abnormal decrease for most cities. The scaling relations of local dimension gradually break if the *q* value is too high. Despite this, the different levels of the network always keep the scaling relations reflecting singularity exponent. The main conclusions can be reached as follows. First, urban street networks follow multifractal scaling law, and scaling precedes local fractal structure. Second, the patterns of traffic network take on characteristics of spatial concentration, but it also shows contradictory signs of the spatial deconcentration. Third, the development space of central area and high-density area is limited, while the fringe zone and low-density area have the phenomena of disordered evolution. This study may be revealing for future complex network research such as urban transportation analysis by using multifractal theory.”

**2.**	All section 2 must be reorganized in a better way. Whenever the author introduce a feature (q, D_q, tau_q definition of multi and mono fractal) is SHOULD be followed but a proper definition. I found some of them only at the end of the section that clarified the parameters. Please, move the AT THE FIRST and EVERY time a new variable appears in the text.

**[Reply]** Section 2 has been reorganized to be more understandable. Each parameter is followed by its definition nearby. All these changes can be found in Line 81-188.

**3.**	Eq.5 N_i(alpha,epsilon) in the equation but right after the subscript 'i' disappears. Please, report what it stands for right before or after the eq. 5.

**[Reply]** We are very sorry for this mistake in Eq (5). The correct expression is $N(\alpha,\varepsilon_{i})\propto\varepsilon_{i}^{-f(\alpha)}$. And $N(\alpha,\varepsilon_{i})$ represents the number of fractal subsets with the same *α* value under the linear size *ε_i_*. We have corrected it in Line 138-142:

“Accordingly, the number of fractal subsets with the same *α* value under the linear *ε_i_* is given by

$N(\alpha,\varepsilon_{i})\propto\varepsilon_{i}^{-f(\alpha)}$, (5)

where *f*(*α*) refers to the fractal dimension of the subsets with singularity exponent *α*, named as *local fractal dimension*.”

**4.**	Fig. 1 ) It might help to put the value of the entropy of the example just to give an idea of the magnitude of different pattern

**[Reply]** Thank you for providing this insight. But we have removed this figure due to the copyright issues (Chen and Wang, 2013). And a figure has been added to explain how the dominant structure of street network changes over the moment order *q* (Fig 2).

- Chen YG, Wang JJ. Multifractal characterization of urban form and growth: the case of Beijing. Environ Plann B. 2013;40(5):884-904. doi: 10.1068/b36155.

**Fig 2. Schematic representation of the filtering procedure for multifractal computation conducted by box-counting method with a regular grid of 16*16 boxes.** The probability distribution of traffic links is calculated over all boxes and then weighted by moment order *q*. Dark red boxes represent dominant structures with higher weighted probability, and dark blue boxes represent lightweight regions with lower weighted probability. (A) Partial traffic networks in Shenzhen. (B) When *q*=0, all non-empty boxes are equally weighted (gray). So the capacity dimension *D*_0_ is a measure of the space-filling capacity. (C) When *q*=1, it reflects the realistic density distribution of traffic networks. (D)-(E) For *q*>0, the boxes with relative high-density measures gradually gain more importance and their contribution to the entropy will dominate. (F) For *q*<0, the boxes with relatively low-density measures gradually gain more importance and their contribution to the entropy will dominate. In this way, with *q* changes from -∞ to ∞ step by step, the multifractal parameter spectrums correspond to urban fringes, main urban areas and central areas.

**5.**	Top pg. 6. What their definition of 'interaction'?

**[Reply]** This paragraph has been deleted now.

**6.**	pg. 7 ) There are some bullets in blue. The author might consider to write them in bold if they want to highlight those sentences.

**[Reply]** We have corrected the text as suggested.

**7.**	What correspond a(0) and f(a(0))... is it q = 0? alpha doesn't appear in the text as function of something. At least not directly. Please, specify.

**[Reply]** Yes, *α*(0) is the value of singularity exponent *α* when *q*=0, and *f*(*α*(0)) is the value of local fractal dimension when *q*=0. We corrected it as “(*α*(*q*=0), *f*(*α*(*q*=0)))” in Line 147.

“It is a convex parabola with an apex (*α*(*q*=0), *f*(*α*(*q*=0))).”

**8.**	pg. 8 ) " reflecting by", please consider the correctness of this sentence.

**[Reply]** We corrected it in Line 194-195. And we have rechecked the grammar of the full text.

“The population size characterized by urban permanent residents is near or even greater than five million for each megacity.”

**9.**	Fig. 4 It results shifted and not well centered in the page. The label of the first row of figure are in the other page. Please, consider to reorder them and report the figure in only one page.

**[Reply]** We have fixed this problem in this revision.

**10.**	Fig. 7. The ticks of all axes are too distanced. please consider to put minor ticks and ticksLabel as well. Moreover the axes label are not clear to me (maybe badly centered?). In addition, the grey points present in all plots are not clear what do they represent. Please consider to put a legend and/or a clearer explanation. But, in general I am not convinced by the goodness of this linear fitting of the scatter plot, starting always for the origin. Do the authors considered to compute the critical *q* when the value in y-axis go from 0 to >0 ?

**[Reply]** Thank you for providing these insights. As suggested, we replaced the fix-intercept linear regression method with the conventional ordinary least square regression for multifractal computation. The original Fig 7 (now Fig 9) was redrawn for better understanding. And a table was added to reflect the statistic thresholds of the moment order *q* based on significance level *α*=0.05 of local dimensions *f*(*q*) (Table 4).

**Table 4. The statistic thresholds of the moment order *q* based on significance level *α*=0.05.**

| Region | City | For *q*<0 | For *q*>0 | Region | City | For *q*<0 | For *q*>0 |
| --- | --- | --- | --- | --- | --- | --- | --- |
| North China | Beijing | -4 | 16 | Central China | Wuhan | -40 | 26 |
|  | Tianjin | -40 | 19 |  | Zhengzhou | -40 | 34 |
| South China | Guangzhou | -40 | 10 | West China | Xi’an | -40 | 11 |
|  | Shenzhen | -40 | 33 |  | Chengdu | -40 | 13 |
| East China | Shanghai | -40 | 16 | Northeast China | Shenyang | -40 | 27 |
|  | Nanjing | -40 | 21 |  | Harbin | -40 | 40 |

**Note:** If the *q* value exceeds the statistical threshold, the confidence level of the local fractal relation will be less than 95%. For example, for Beijing, if *q*<-4 or *q*>16, the local fractal relation will be not significant at the level *α*=0.05.

**Fig 9. The log-log plots for estimating the local fractal dimension *f*(*q*) with changes of *q*.** With the absolute value of *q* increases, the scattered points in log-log plots become more and more disordered, and the scaling relationships are broken seriously.

We agree that the fix-intercept regression method may not be suitable enough here. The OLS regression has two different approaches. One is to fix the intercept to zero, and the other is conventional free-intercept regression. According to Huang and Chen (2018), the zero-intercept regression can yield standard multifractal parameter spectrums. While the common regression results may show some abnormal curves, which suggest problems in urban structure. So the latter will be more suitable for empirical analysis.

- Huang LS, Chen YG. A Comparison between Two Ols-Based Approaches to Estimating Urban Multifractal Parameters. Fractals. 2018;26(1). doi: 10.1142/S0218348x18500196.

Moreover, as suggested by reviewers, we chose to take the street links as our measurements. Because street links may provide more information about local connectivity, which can better represent the network density. As a reference, we also provide the multifractal computation results generated by street nodes (see S2 Appendix). Results show that the value of some multifractal parameters changed to some extent (Table 2 and 3). And the analysis results and conclusions have been adjusted accordingly.

We found that the scaling relations of singularity exponent *α*(*q*) maintain well with *q* changes (Table 3). However, the scaling relations of local fractal dimension *f*(*q*) degrades when *q* approaches infinity. The goodness of fit of *f*(*q*) decreases seriously, and the scattered points in the double logarithmic plot (log-log plot) become more and more chaotic correspondingly (Fig 9). This suggests that fractal dimension is the strictest scaling exponent.

The significance test was made to multifractal parameters by the OLS regression method. Table 4 summarizes the statistic thresholds of the moment order *q* based on significance level *α*=0.05 of local dimensions *f*(*q*). The different levels of a self-similar hierarchy system can be reflected by the moment order *q*. Results show that structural levels vary greatly among cities. Generally, the range of *q*>0 is narrower than that of *q*<0, indicating worse fractal structure in central areas.

We have added the following discussion on this issue in our revised manuscript, see Line 348-353 and Line 360-372.

“The urban street network follows the scaling law in different parts and different levels, but the local fractal feature is not equivalent to the scaling relation. The scaling relations representing singularity exponent *α*(*q*) maintain well with the changes of moment order *q*. However, the fractal relations of local fractal dimension *f*(*q*) display partial degradation. The degradation of scaling relation means that the power law describing this scaling relation is broken, and the scatter points on the corresponding log-log plot are distributed disorderly, rather than with a linear trend (Fig 9).”

“In sparse areas and marginal areas, the urban street network presents disorderly development, while in dense areas and central areas the fractal structure degenerates gradually. As mentioned above, when *q* approaches negative infinity, the multifractal spectrums gradually focus on the low-density areas. Conversely, when *q* approaches positive infinity, the multifractal spectrums gradually focus on high-density areas. On the one hand, when *q* approaches negative infinity, the generalized correlation dimension significantly breaks through the Euclidean dimension of the embedding space, which is *D*(*q*) >> *d* = 2. And the local fractal dimension *f*(*q*) curve shows an abnormal decrease for most cities. These disorders may be related to the poor development and fragmentation construction of street networks in sparse areas and urban fringes. On the other hand, when *q* approaches positive infinity, the scaling relations of *f*(*q*) present a universal degradation at a certain level. This reflects the street networks in central areas have been constructed in a highly compact way, leaving limited free space. Different parts of multifractal spectrums reflect the characteristics of different levels of urban street networks.”

Special thanks to you for your good comments.

### 2.2 Reviewer #2:

The article proposed the method to test the urban network in 12 megacities in China to see whether the multifractal structure can be observed. Then the multifractal analysis is applied to gain the spatial characteristics of the urban network. The results indicate that the street networks have significant multifractal structures and also has the spatial heterogeneity and hierarchical structure.

From the paper, the following comments are provided:

1. The article has new data and used in a well-structured method, but there is not much clarification of the contribution which needs to elaborate further. In the conclusion part, it says by knowing the multifractal structure of the urban network, the accessibility of the urban system can be improved via the optimization, but how it would be guided through this proposed method and the contribution of this article needs to be specified.

**[Reply]** Several lines of words were added to the text to explain the function of multifractals in spatial optimization (Line 373-377):

“The gap between the development goal and the status quo of a system is just the problem. The process of solving problems is the process of geographic system optimization. The important function of multifractal in geographical practice is the diagnosis of spatial problems. Only when the problem is made clear can the problem be solved, so as to achieve the aim of optimizing the geographical space.”

2. Page 8, line 17: “Nevertheless, compared with street lines, the distribution of street nodes has been more stable over times.” The first thing is ‘times’ should be ‘time’ here. Some references needed to support this statement. I realize this issue has been re-raised in the Discussion part, so a general suggestion is to reframe this part to show a more precise illustration.

**[Reply]** Thanks for pointing this out. Referring to Murcio *et al.* (2015), we originally chose the street nodes distribution as the representative of street network distribution. In this revision, as suggested by reviewers, we replace street nodes with street links as our measurements. Because street links may contain more information about local connectivity, which better reflect the network density. Results show that the value of some multifractal parameters changed to some extent (Table 2 and 3). And the analysis results and conclusions have been adjusted accordingly.

- Murcio R, Masucci AP, Arcaute E, Batty M (2015). Multifractal to monofractal evolution of the London street network. Physical Review E, 92, 062130

We have added the following discussion on this issue in our revised manuscript, see Line 223-226.

“Then we conduct multifractal computation for each one of 12 urban street networks. In contrast to Ref [1], we extract street links for analysis instead of street nodes. Because street links may contain more information about local connectivity, which better reflect the network density. As a reference, we also provide the multifractal computation results generated by street nodes (see S2 Appendix).”

3. Page 17: the discussion about the population distribution seems irrelevant to this part.

**[Reply]** We have removed this and reorganized the Discussion section.

4. Page 20: “The single scaling process leads to mono-fractal structure, showing a uniform distribution. While multiple scaling process lead to multifractal structure.” This statement is confusing and irrelevant to the first part of the conclusion. I inferred that whether the system is monofractal or multifractal merely based on model selection. Thus, it weeks the conclusion of the multifractal would be better to suit the real-world complexity.

**[Reply]** Several statements about multifractal and monofractal structure were more ambiguous than intended. A fractal can be defined as a shape made of parts that are similar to the whole in some way (Feder,1988), with the characteristic of scale-invariant. It is easy for geographers to understand the concept of scaling, because the process of zooming in or out of a map is a typical scaling process. For a monofractal object, it has only one scale factor. That is to say, if the local of fractal element is enlarged to make it the same as the whole, or the whole is reduced to make it the same as the local, only a scaling ratio is needed.

Simple models help understand complex structure. We construct two types of regular fractal structure by scaling ratio in the following figure. For a monofractal object (see Fig A1), it develops with the same probability at different grow points, and with the same rate in different directions which are orthogonal (Jullien *et al*, 1987; Vicsek, 1989). Accordingly, its density distribution is uniform, and can be characterized by a single global dimension. This model is generated by a single scaling ratio. In fact, multifractal is also called multi-scaling fractals, which contains two or more scaling process. In Fig A2 and A3, we construct two kinds of multifractals by two different scale ratios. They have different growth probabilities in various parts, leading to a heterogeneous distribution (Chen, 2019; Murcio et al.,2015). The difference between monofractals and multifractals are illustrated in Table A.

- Feder J (1988). *Fractals*. New York: Plenum Press
- Jullien R, Botet R (1987). Aggregation and fractal aggregates. Singapore: world Scientific Publishing
- Vicsek T (1989). Fractal Growth Phenomena. Singapore: World Scientific Publishing
- Chen YG (2019). Monofractal, multifractals, and self-affine fractals in urban studies. Progress in Geography, 38(1): 38-49 [In Chinese]
- Murcio R, Masucci AP, Arcaute E, Batty M (2015). Multifractal to monofractal evolution of the London street network. Physical Review E, 92, 062130
- Chen YG, Wang JJ (2013). Multifractal characterization of urban form and growth: the case of Beijing. Environment and Planning B: Planning and Design, 40(5): 884-904

**Fig A.** The first four generation steps of two types of regular growing fractals: monofractal and multifractals. The area of the square represents growth probability or density of different subsets. A1 is monofractal, in which every subset shares the same growth probability. While A2 and A3 are multifractals, in which different subsets have different growth probabilities. According to distinct dynamics of spatial-temporal evolution, they can be distributed into two typical classes: spatial concentration A2 and spatial deconcentration A3.

Table A. Comparison between monofractals (simple fractals) and multifractals (complex fractals)

| **Item** | **Monofractals** | **Multifractals** |
| --- | --- | --- |
| **Form/pattern** | Self-similarity | Self-similarity and self-affinity |
| **Growth** | isotropy | isotropy |
| **Developing chance** | equal probability at different  directions and parts | inequal probability at  different parts |
| **Dimension** | single dimension value | spectrums of fractal  dimension values |
| **Scaling** | monoscaling process | multiscaling processes |

Therefore, a monofractal has one scaling process, while multifractals have two or more scaling processes. However, for random distributions, it is difficult to distinguish one from another. For a city, we can estimate its fractal dimension values by using the box-counting method, and then judge whether or not the urban form is a monofractal or multifractal phenomenon (Chen and Wang, 2013). In the real world there exists many other systems which have different growth probabilities at different parts and non-uniform density distribution. They can be treated as multifractals instead of monofractals. In urban geography, multifractal properties have been demonstrated in urban population (Appleby, 1996, Semecurbe *et al*., 2016), urban land use (Chen and Wang, 2013, Man *et al*., 2019), land price (Hu *et al*., 2012, Salat *et al*., 2018). And Frankhauser, P., *et al*. make efforts to apply the multifractal modeling to urban or regional planning (Frankhauser, P., *et al.,*2018; Yamu, C. and Frankhauser, P.,2015; Yamu, C. and Nes, A.,2017). This paper is devoted to exploring the multifractal structure of urban traffic networks.

- Appleby S (1996). Multifractal characterization of the distribution pattern of the human population. Geographical Analysis, 28(2): 147-160
- Semecurbe F, Tannier C, Roux SG (2016). Spatial distribution of human population in France: Exploring the modifiable areal unit problem using multifractal analysis. Geographical Analysis, 48: 292-313
- Man W, Nie Q, Li ZM, Li H, Wu XW (2019). Using fractals and multifractals to characterize the spatiotemporal pattern of impervious surfaces in a coastal city: Xiamen, China. Physica A: Statistical Mechanics and Its Applications, 520: 44-53
- Hu SG, Cheng QM, Wang L, Xie SY (2012). Multifractal characterization of urban residential land price in space and time. Applied Geography, 34: 161-170
- Salat H, Murcio R, Yano K, Arcaute E (2018). Uncovering inequality through multifractality of land prices: 1912 and contemporary Kyoto. PLoS ONE, 13(4): e0196737.
- Frankhauser, P., Tannier, C., Vuidel, G. & Houot, H. (2018). An integrated multifractal modelling to urban and regional planning. Computers Environment and Urban Systems, 67, 132-146.
- Yamu, C. & Frankhauser, P. (2015). Spatial accessibility to amenities, natural areas and urban green spaces: using a multiscale, multifractal simulation model for managing urban sprawl. Environment and Planning B-Planning & Design, 42, 1054-1078.
- Yamu, C. & Nes, A. (2017). An Integrated Modeling Approach Combining Multifractal Urban Planning with a Space Syntax Perspective. Urban Science, 1, 37.

In this revision, a paragraph was added to further explain the differences between multifractals and monofractals. Please refer to Line 82-93:

“Monofractal is the simplest fractal structure, widely employed to explain urban morphology and growth. The fractal dimensions are computed by the distribution density of a specific measure in systems. For a self-similar monofractal object, it develops with the same probability at different grow points, and with the same rate in different directions which are orthogonal [31]. When a monofractal system is characterized by a single global dimension, it is implicitly assumed that its density distribution is uniform, and the growth probability is equal in different regions. However, in the real world there exist many other systems whose density distribution is non-uniform, and sometimes presenting discontinuities at different scales [1, 20]. They can be treated as multifractals instead of monofractals, especially for cities. Traffic network system is a large system embedded in the geographical space, the spatial growth of which contains lots of local subsystems. They have different growth probabilities and scaling processes, leading to a heterogeneous distribution. So multifractal analysis may be preferable.”

5. The phrase ‘asymmetric cascade structure’ can only be found in abstract while no other place in this article has mentioned this concept.

**[Reply]** Now, this concept has been linked to Line 333-229.

“The multifractal network is a kind of self-similar hierarchy with asymmetric cascade structure. The different levels of a self-similar hierarchical system can be reflected by the moment order *q*. Where global level is concerned, changing *q* value indicates changing described levels of multifractal system; where local level is concerned, changing *q* value indicates changing focused parts in the multifractal system. One of the advantages of multifractal method is that, by means of multi fractal dimension spectrums, different levels of a complex network can be investigated at the macro level, and different parts can be focused at the micro level.”

6. The paper has too many minor grammar errors: missing articles; the plural form is not consistency, like Page 3 line 10: ‘has’ should be ‘have,’ line 11 ‘method’ is not agree in number with other words; Page 3 line 8: ‘meterials’ should be ‘materials,’ follows the ‘founctional’ do you mean ‘functional’? I urge another round of proofreading is necessary.

**[Reply]** We apologize for these grammatical errors. We have fixed these errors and rechecked the grammar of the full text.

7. Some sentences causing frustrations need to reconsider:

Page 1,line 4: “cities, systems of cities, and traffic networks” seems not parallel concepts;

Page 8,line 1: “twelve Chinese megacities in China…” is redundant

Page 8, line 5: “As these cities…” this sentence lack logic.

**[Reply]** Thank you for pointing out these problems. We have corrected these sentences.

We agree that “cities, systems of cities, and traffic networks” are not parallel concepts. But they are different complex systems in different fractal studies.

Line 35-37:

“It has been widely employed to characterize complex systems, such as river networks, systems of cities and traffic networks, and to reveal their spatial patterns and scaling properties.”

Line 191:

“In this work, twelve Chinese megacities are selected for case studies of traffic networks.”

Line 195-196:

“In these cities, human activities and urban flows are highly concentrated on street networks.”

We acknowledge your comments and suggestions very much, which are valuable in improving the quality of our manuscript.

### 2.3 Reviewer #3:

The paper conducts an analysis street networks in 12 mega-cities in China, based on the assumption that they are multifractal systems. Overall, considering the conclusions of the study, the idea seems to be an extreme over-complication of the problem at hand while the framework has major weaknesses. I strongly believe authors have failed to establish the necessity and rigorousness of their analysis over widely-used network analyses.

**[Reply]** Dear reviewer, please note two facts. First, multifractal theory is complicated, but the results from multifractal spectrums are simple. Second, multifractals and hierarchical networks share the same heterogeneous structure of complex systems.

Multifractal theory does be an excellent mathematical tool for geographical spatial analysis. Multifractal scaling implies spatial heterogeneity, and different parts have different fractal dimensions and different scaling exponents. It is difficult to describe the various parts in a heterogeneous system. Using multifractal methods, we can transform the spatial features of different parts into multifractal spectrums by means of the moment order *q*. Thus, complex spatial structure become several simple spectral curves. Through the spectral curves, we can examine the different parts and different levels of a geographical system. In this sense, multifractal spectra are compared to telescopes and microscopes. Unfortunately, it is difficult to understand the basic principle of multifractal geometry. There is no shortcut to learning this mathematical tool. The popularization of multifractal analysis depends on the development of computer programs. However, before making computer programs for multifractal analysis in urban geography, we need to carry out a lot of research to reveal the application direction of multifractal method.

Multifractals is related to scale-free network, and can be used to describe the heterogeneous structure of hierarchical network. The following table displays the relationships between fractals and self-organized networks. The scaling exponent of a hierarchical network *b* is equal to fractal dimension *D* plus 1, i.e., *b*=1+*D*.

**Table 5. Relationships between self-organized network and fractal structure.**

| Type | Network | Degree distribution | Cluster coefficient | Fractal structure |
| --- | --- | --- | --- | --- |
| Simple network | Random network | Poisson (exponential) | Uniform | No |
|  | Small world | Poisson (exponential) | Uniform | No |
| Complex network | Scale-free network | Power law | Uniform | Related |
|  | Hierarchical network | Power law | Power law | Yes |

**Note**: The contents in this table are summarized by referring to Barabási (2002), Barabási and Albert (1999), Barabasi and Bonabeau (2003), etc.

Major comments:

1.The paper is not well-written and there is an abundance of errors and hastily written sentences. The second paragraph in Introduction has a sum of more than a dozen grammatical and spelling and writing issues. Many sentences in the text are very ambiguous and convey no specific message and need to be properly explained or removed from the paper. Examples include but they are not limited to those below, taken from the beginning of the paper:

- It is difficult to model and analyze complex networks by conventional mathematical methods.

- The radial dimension proved to be a special spatial correlation dimension of fractals.

- Multifractal method bears analogy with telescopes and microscopes in geographical spatial analysis.

**[Reply]** Thank you for pointing out these problems. To avoid misunderstanding, we have removed some sentences and rechecked the grammar of the full text.

Dear reviewer, all the following statements are easy to understand if you are familiar with multifractal theory.

- It is difficult to model and analyze complex networks by conventional mathematical methods. [In fact, conventional mathematical methods depend on characteristic scale, but complex networks bear no characteristic scale in many aspects. Multifractal geometry is a good method for scale-free analysis.]

- The radial dimension proved to be a special spatial correlation dimension of fractals. [In fact, multifractal dimension spectrum includes capacity dimension, information dimension, and correlation. The radial dimension was proved to be a kind of correlation dimension. See the following book and paper, you will understand this sentence: Takayasu H. *Fractals in the Physical Sciences*. Manchester: Manchester University Press, 1990; Chen YG. Fractal analytical approach of urban form based on spatial correlation function. *Chaos, Solitons & Fractals*, 2013, 49(1): 47-60. About the concept of radial dimension please see: Frankhauser P, Sadler R. Fractal analysis of agglomerations. In: *Natural Structures: Principles, Strategies, and Models in Architecture and Nature*. Ed. M. Hilliges. Stuttgart: University of Stuttgart, 1991, pp 57-65]

- Multifractal method bears analogy with telescopes and microscopes in geographical spatial analysis. [In fact, multifractal method can be used to scan and magnify geographical phenomena. The function of multifractal is similar to wavelet, which is regarded as a telescope and microscope in mathematical tools.]

We have added some interpretations for this sentence. Please refer to Line 51-55:

“Multifractal method bears analogy with telescopes and microscopes in geographical spatial analysis, which takes into account the relative intensity related to the particular zones of the city. It provides a series of parameter spectrums adequately capturing the spatial heterogeneity of fractal patterns and the statistical distribution of measurements across spatial scales [19, 20].”

2.A major weakness of the paper is its inability to deliver a clear and effortless description of the framework. Also, procedures such as “city clustering algorithm” and “head-tail breaks” are not described in the paper; especially the former is not at all widely-used and needs clear explanation.

**[Reply]** In this revision, we have reorganized the Introduction section to clarify the framework of our paper. Please refer to Line 70-80. And we have added a paragraph to explain the details of identifying urban boundary by city clustering algorithm (CCA) and head-tail breaks (Line 197-210).

“This study has two main objectives: (1) to examine whether the traffic networks of China generally have multifractal scaling characteristics. (2) to decipt the spatial patterns of the whole USND, especially differences between the central and fringe areas, and determine the dominant multifractal complexity characteristics within cities.

The remainder of this article is organized as follows. In Methodology section, the multifractal method and related two sets of parameters are introduced. Empirical Results section details the study areas and main results of multifractal analysis. The global multifractal spectrums reflect the overall spatial patterns of USND. And the local multifractal spectrums provide local and detailed characteristics. In Discussion, the key points of analyzed results are outlined, and related questions are discussed. Finally, the discussion is concluded by summarizing the main inferences of this work.”

“Considering that different definitions of cities may affect conclusions regarding the statistical distribution of urban activity [40], we first identify the urban boundary in a consistent way as our study area. Many algorithms have been constructed to delimit urban boundaries [41-44]. Especially, City Clustering Algorithm (CCA) has attracted great attention for its simplicity and efficiency [45-47]. CCA defines cities as the maximally connected populated clusters within a prescribed distance, and later it has been expanded to the spatial clustering of massive street nodes [48]. Our data processing includes three main steps. First, street network datasets in 2016 are obtained from the Chinese digital navigation map (http://geodata.pku.edu.cn), including freeways, arterials, and collectors. Based on this, we then derive 6.74 million street nodes by ESRI ArcGIS. Second, we apply the CCA to cluster contiguous nodes by using the Aggregate Points tool in ArcGIS, which is intrinsically based on a TIN (Triangulated Irregular Network) model. In this process, the aggregation distance is set as 615 meters, which is the mean length of whole TIN edges determined by head-tail breaks [49]. And it’s very close to the best resolution found in Ref [48]. Third, we fill the holes inside the clusters and select the largest cluster of each city as identified urban boundary (Fig 4).”

3. There is a need for at least present a synthetic example of how a non-fractal network system would compare to those analysed in this paper. Perhaps by generating a network with insignificant fractality and then performing comparisons by means of D0, D1, and D2.

**[Reply]** Nonfractal systems have no fractal dimension. Fractal geometry can only be applied to fractal systems. Random network, small world, etc., are well-known non-fractal network. Hierarchical network are fractal network or complex network associated with fractals. All these knowledge can be found in literature on self-organized networks. Due to the limitation of the paper’s space, we cannot discuss these questions in too more words. See for example:

- Barabási A, Albert R. Emergence of scaling in random networks. Science, 1999, 286: 509-512
- Barabási A-L. *Linked: The New Science of Network*. Cambridge: Persus Publishing, 2002.
- Barabasi A-L, Bonabeau E. Scale-free networks. *Scientific American*, 2003, 288(5): 50-59

A table has been added to the text as below, showing the connection and differences between network structure and fractals:

**Table 5. Relationships between self-organized network and fractal structure**

| Type | Network | Degree distribution | Cluster coefficient | Fractal structure |
| --- | --- | --- | --- | --- |
| Simple network | Random network | Poisson (exponential) | Uniform | No |
|  | Small world | Poisson (exponential) | Uniform | No |
| Complex network | Scale-free network | Power law | Uniform | Related |
|  | Hierarchical network | Power law | Power law | Yes |

Scale-free network can be associated with fractals, and hierarchical network include fractal structure. And we have added the following discussion on this issue in Line 379-387:

“We agree with Batty [51], who once pointed out: “An integrated theory of how cities evolve, linking urban economics and transportation behavior to developments in (self-organized) network science, allometric growth, and fractal geometry, is being slowly developed.” Fractal structure proved to be a type of spatial order emerging at the edge of chaos [52-55]. By using fractal geometry, we can combine urban transportation behavior with urban land use pattern and complex network structure. Hierarchical network includes fractal structure, and scale-free network can be associated with fractals (Table 5). The commonness of complex network and fractals lies in scaling [51, 56-58]. Fractal geometry provides an efficient approach for scaling analysis. ”

4. Significance of the parameters need to be tested, for example via determining the same parameters in well-studied structures with high, moderate, or no fractality.

**[Reply]** In this revision, the goodness of fit (*R*^2^), the *p*-value, and the standard error of each parameter are given in Table 2, Table 3, and S3 Table.

Dear reviewer, as a matter of fact, the significance of the parameters has been tested by *R* square (*R*^2). In this study, the *R* square can be used to reflect goodness of fit, *F* statistic, *t* statistic, and standard error. The fractal model parameter estimation is in fact based on one variable logarithmic linear regression. In this case, the goodness of fit (*R*^2^) is equivalent to *F* statistic (at the macro level) and *t* statistic (at the micro level), and can be associated with standard error. This question was discussed by Chen (2015, 2018) as follows:

“Different coefficients of determination result in different values of *F* statistic, *t* statistics, and standard errors. The *F* statistics can be used to judge the cause and effect, the *t* statistics can be utilized to judge the level of confidence of a model’s parameter, and the standard errors can be employed to estimate the margin of error of a coefficient. For the regression analysis with a single explanatory variable, the *F* statistic, *t* statistics, and parameter standard errors (*δ*) are all equivalent to the *R* square, and can be computed by the following formulae

$F=t^{2}=\frac{vR^{2}}{1-R^{2}}$, (39)

$\delta=R_{c}\sqrt{\frac{1}{v}(\frac{1}{R^{2}}-1)}$, (40)

where *v* refers to the residual degree of freedom.”

“By the knowledge from statistics, we can derive the relation between the standard error and correlation coefficient in the form

$\delta=D\sqrt{\frac{1/R^{2}-1}{n-2}}$. (12)

This suggests that, for given fractal dimension *D* value, the standard error *δ* is equivalent to the goodness of fit, *R*^2^ values. However, the *R*^2^ value is a statistic for description, not for inference.”

- Chen YG (2015). A new methodology of spatial cross-correlation analysis. *PLoS ONE*, 10(5): e0126158
- Chen YG (2018). Logistic models of fractal dimension growth of urban morphology. *Fractals*, 26(3): 1850033

See the response to the related comments of reviewer #4.

The function of model parameters (e.g., scaling exponent, fractal dimension, regression coefficient) differs from that of statistic parameters (e.g., Pearson correlation coefficient, student’s *t* statistic) for testing. Model parameters are quantities for inference, while statistic parameters are quantities for description. We use the fractal dimensions to make inferences, while use statistic parameters to evaluate the effect of modeling building.

5. A significant issue is what appears to be, ignoring the links (representing road segments) and focusing on the network nodes, when analysing the road networks. This can be immensely misleading, especially in a study like this where the goal is (at least to great extent) to characterize the network connectivity. Density of nodes which determines the parameters found this study, has actually little to do with local connectivity. A large number of connected nodes with few connections make a sparse network. This is the reason that network density (as a connectivity index) is measured by the number of network links.

**[Reply]** Thank you for providing these insights. We agree that street links may better reflect local connectivity of urban street networks. In this revision, we took the street links as our measurements as suggested. And we also replaced the fix-intercept linear regression method with the conventional ordinary least square regression for multifractal computation. The OLS regression has two different approaches. One is to fix the intercept to zero, and the other is conventional free-intercept regression. According to Huang and Chen (2018), the zero-intercept regression can yield standard multifractal parameter spectrums. While the common regression results may show some abnormal curves, which may suggest some problems in urban structure. So the latter is more suitable for empirical analysis.

- Huang LS, Chen YG (2018). A comparison between two OLS-based approaches to estimating urban multifractal parameters. Fractals, 26(1): 1850019

We have added the following discussion on this issue in our revised manuscript, see Line 223-231.

“Then we conduct multifractal computation for each one of 12 urban street networks. In contrast to Ref [31], we extract street links for analysis instead of street nodes. Because street links may contain more information about local connectivity, which better reflect the network density. As a reference, we also provide the multifractal computation results generated by street nodes (S2 Appendix). The functional box-counting method is applied to extract basis data for fractal parameter estimation (S1 Table). The scale range of spatial subdivision is set as 2^0^~2^9^. Besides, the value range from -40 to 40 is selected for *q*, as multifractal parameters are very close to their convergence when $\left| q \right|$ approaches 40 (S2 Table). The multifractal parameters are calculated by the OLS linear regression (S3 Table).”

The calculation results and analysis have been updated in our revision.

Minor comments:

6. From the paper: “If an urban system bears multifractal structure, then different subareas have different growth probabilities and density distribution.” Based on the above statement, can we conclude non-multifractal structure indicate similar growth and density distributions? If yes, which I believe is incorrect, then authors should explain why. And if not, then there always disadvantage in using a complex analysis to characterize a simple property, and authors better provide why their framework is any different.

**[Reply]** Dear reviewer, we only use multifractal geometry to describe and study multi-scaling fractal phenomena. We never apply multifractal theory and method to monofractal phenomena and non-fractal phenomena. To our thinking, one method should be used properly. A comparison between non-fractal, monofractal, and multifractals can be drawn as follows (see the table below).

| Type | Distribution | Example | Density distribution | Spatial feature |
| --- | --- | --- | --- | --- |
| Non-fractal (three typical nonfractal phenomena) | Uniform distribution | Square grid | Absolute uniformity | Homogeneity + characteristic scale |
|  | Random distribution | Poisson distribution | Statistic uniformity | Homogeneity + characteristic scale |
|  | Spatial decay with characteristic length | Normal distribution | Circular uniformity | Homogeneity + characteristic scale + distance decay |
| Monofractal | Simple self-similar distribution | Sierpinski gasket, Vicsek carpet | All the fractal units are similar to one another | Homogeneity + single scaling |
| Multifractals | Complex self-similar distribution | Vicsek growing multifractal | A fractal unit may differ from another fractal unit | Heterogeneity + multiple scaling |

We hope this explanation could address your concerns with our work.

7. Try to reduce the usage of “;” when it is more appropriate to break the sentence in two; in many cases it is used inappropriately.

**[Reply]** Thanks for your kind suggestions. In this revision, we removed “;” and adjusted to our text.

8. Although one might be able to guess based on the context, it is unclear what “if D0>D1>D2 significantly” means.

**[Reply]** Dear reviewer, “significantly” is an expression of statistics. In statistics, we never say a parameter is zero, instead, we say the parameter has no significant difference from zero, or, we say the parameter is not significantly different from zero. A significance level (e.g., *α*=0.05) corresponds to a level of confidence ((1-*α*)*100%=95%). The significance level is forever a relative concept (*α*=0.15, 0.1, 0.05, 0.01, 0.005, 0.001, etc.).

If *D*_0_-*D*_1_, i.e., the difference between *D*_0_ and *D*_1_, is proved to be different from zero based on the significance level *α*=0.05, we will say we have a confidence level of 95% to believe that *D*_0_ is greater than *D*_1_.

If *D*_0_-*D*_1_ is proved to be different from zero based on the significance level *α*=0.01, we will say we have a confidence level of 99% to believe that *D*_0_ is greater than *D*_1_.

The rest may be deduced by analogy.

For simplicity, we say in general that “if *D*_0_>*D*_1_>*D*_2_ significantly”.

9. It is hard to understand how this sentence helps the logical flow in interpreting the fractality of urban traffic networks: “Where there is interaction in a geographical region, there is an urban system, and where there is an urban system, there is a traffic network.”

**[Reply]** If it is difficult to understand for readers, this statement can be removed.

An urban system is based on interaction, which in turn depends on traffic network. In the field of urban geography, the concept of urban system is controversial. In fact, the urban system can be defined by spatial interaction. Spatial interaction is based on transportation and communication networks.

10. The goal is really obscured when sections 2.1 is presented before 2.2. I suggest authors mix the two somehow, or try to involve more interpretation when presenting the abstract ideas of multifractality.

**[Reply]** Thanks for your kind suggestions. Several statements about multifractal were more ambiguous than intended, and we adjusted to the text to be clear. Please refer to Line 82-93:

“Monofractal is the simplest fractal structure, widely employed to explain urban morphology and growth. The fractal dimensions are computed by the distribution density of a specific measure in systems. For a self-similar monofractal object, it develops with the same probability at different grow points, and with the same rate in different directions which are orthogonal [30]. When a monofractal system is characterized by a single global dimension, it is implicitly assumed that its density distribution is uniform, and the growth probability is equal in different regions. However, in the real world there exist many other systems whose density distribution is non-uniform, and sometimes presenting discontinuities at different scales [19, 31]. They can be treated as multifractals instead of monofractals, especially for cities. Traffic network system is a large system embedded in the geographical space, the spatial growth of which contains lots of local subsystems. They have different growth probabilities and scaling processes, leading to a heterogeneous distribution. So multifractal analysis may be preferable.”

11. Figure 2 is never pointed out in the text. I think the reference to Fig. 3 in page 7 needs correction.

**[Reply]** We apologize for this mistake. The original Fig 2 (now Fig 1) has been linked to Line 149.

12. I believe authors should not write sentences in “bold,” in order to highlight their importance. Try to highlight the important points using appropriate and effective writing.

**[Reply]** We have adjusted to the text as suggested.

13. “Good order” as opposed to “disorder” should be explained clearly in concept and how it can be interpreted from the planner or user perspective. The way these abstract concepts appears in the paper, is not informative to the reader and only appears as unnecessary jargon here and there, only making the text hard to follow.

**[Reply]** The expressions have been revised now, so that the future readers will understand our meanings readily. In fact, the word “order” indicates the state that exists when things obey natural laws or scientific rules. Otherwise, things are regarded as “disorder”.

In the past, order and disorder were generally discussed in literature. Today, we can find quantitative criteria from multifractal theory to distinguish from order state from disordered state. In this study, we have a set of criteria to judge the state of a fractal system such as a city or a traffic network.

First, multifractal dimension parameters in this paper are measured and calculated by using box-counting method. The box dimension values must come between topological dimension (*d*_T_ =0) and Euclidean dimension of embedding space (*d*_E_=2) in which a fractal system exists. If fractal dimension values exceed the range from *d*_T_=0 to *d*_E_=2, the fractal systems can be treated as “disorder” to a degree.

Second, the spectrum of the generalized correlation dimension, *Dq*, is an inverse S-shaped monotone decline curve. That is, *D*_0_≥*D*_1_≥*D*_2_. If *D*_0_<*D*_1_<*D*_2_. The fractal system can be regarded as “disorder” to some extent.

Third, goodness of fit in different level reflected by the moment order *q*. If the scaling relationship between the linear size of boxes and the probability measures degenerates with the increase (*q*→∞) and decrease (*q*→-∞) of moments, and the goodness of fit decreases significantly, we say that the spatial order becomes worse.

We acknowledge your comments and suggestions very much, which are valuable in improving the quality of our manuscript.

### 2.4 Reviewer #4:

This paper investigates the multifractal scaling laws of urban traffic networks in 12 large Chinese cities. The study uses empirical data to show the existence of such laws. The paper is well-written. I have the following issues with the paper:

1. An important aspect in my opinion is that the findings should be related to some intuition and implications. In its current form the manuscript lack both. I am not sure what the intuition of the three dimensions is for the traffic network, traffic performance, etc. What do I learn from this analysis from D1, D2, and D3? Their three points in the conclusions are a good start, but I am not sure they are completely translated into traffic.

**[Reply]** A new table has been added to the text to explain the three basic fractal dimension, *D*_1_, *D*_2_, and *D*_3_**.**

**Table 1. Geographical meanings of capacity dimension, information dimension, and correlation dimension for traffic network**

| Parameter | Basic measurement | Spatial meaning |
| --- | --- | --- |
| Capacity dimension *D*_0_ | Space-filling degree | Whether or not a place (box) bears elements of networks |
| Information dimension *D*_1_ | Degree of spatial uniformity | How many network elements appear at/in a place (box) |
| Correlation dimension *D*_2_ | Degree of spatial dependence | If a place bears network elements, how many other network elements can be found within certain distance from the place (box) |

**Note**: The degree of spatial uniformity and spatial difference represents the two different sides of the same coin, and spatial difference indicates spatial heterogeneity. The degree of spatial dependence suggests spatial complexity.

2. Similarly, the objective of the study is unclear to me. I believe that clearly stating the research gap and the objective would be emphasize the contributions of the work.

**[Reply]** In this revision, we reorganized the Introduction section (Line 57-73).

“Multifractal theory has been applied to human geography especially, urban geography, for a long time [21-29]. However, there are few reports on multifractal research of traffic networks, such as urban street network. A multifractal system is in fact a self-similar hierarchy with asymmetric cascade structure. Hierarchy and network structure represent two different sides of the same coin [2]. In many cases, it is hard to model network structure mathematically, but it is relatively easy to model hierarchy using proper mathematical tools. In this regard, multifractal theory may provide an advisable approach to studying self-organized complex networks such as urban transportation through self-similar hierarchical networks. To date, few studies have been able to draw on any systematic research into the multifractal structure of traffic networks.

Therefore, this paper is devoted to exploring the multifractal structure of urban street network represented by street links embedded in the two-dimensional Euclidean space. The street networks of twelve typical megacities of China are taken as examples. Chinese digital navigation map in 2016 is collected as materials, and the functional box-counting method is applied to calculate multifractal parameters. This study has two main objectives: (1) to examine whether the traffic networks of China generally have multifractal scaling characteristics. (2) to decipt the spatial patterns of the whole urban street networks, especially differences between the central and fringe areas, and determine the dominant multifractal complexity characteristics within cities. ”

And we have rewritten the Discussion section to emphasize the contributions of our work. Please refer to Line 394-411:

“Compared with the previous studies on urban street networks, especially, the fractal street networks, the novelty of this work lies in the following aspects. First, comprehensive empirical analysis of multifractal scaling in urban street networks. We selected 12 megacities in China as examples, and then defined the study areas in light of the identical standard so that the results are comparable. Through the calculation results of these cities, we can see the general features of multifractal urban street networks. Second, global analysis and local analysis of multifractal structure of urban street networks. We utilize the global parameters to reflect the similarity of different street networks, and use the local parameters to reveal both similarities and differences of these networks among cities. The commonness reflects the law of city development, while the differences may reflect the problems to be solved in urban traffic networks. Third, visual analysis of scaling evolution of local levels of urban traffic networks. We make use of log-log scatterplots to show how the scaling relation of local levels of street networks changes over the moment order *q* values. One of revealing findings is that the local scaling relation reflecting the singularity exponent maintain well across different levels, but the scaling relation representing local fractal dimension may break due to the *q* values are too high or too low. This suggests that fractal dimension is the strictest scaling exponent, and the scaling development difference between the singularity exponent and local fractal dimension can be employed to disclose the fractal structure evolution stages.

3. Sections Discussion and Conclusions are very redundant. I advise the authors to merge these sections.

**[Reply]** In this revision, we have rewritten the Discussion and Conclusions sections. In the Discussion, the key points of analyzed results are outlined, and the contributions of our work are outlined. Finally, the discussion is concluded by summarizing the main inferences of this work.

4. The wording in the manuscript needs substantial revision for consistency. For example, D2 is referred as the correlation dimension; later it’s the connectivity (p10). There are many more examples, where terms are mixed and used interchangeably. I also advise the authors to use commonly used terms such as polycentric, monocentric etc. The term centripetal seems made up in this context. I assume street lines refer to links in a network. Please use the commonly used terms from network science, edges and vertices, or links and nodes.

**[Reply]** We apologize for these errors and have corrected them in this revision.

5. Introductory figures 1 and 2 are a good idea, but they should also be put into the context of traffic networks. Could the authors give an example?

**[Reply]** Thanks for your helpful suggestions. In this revision, a figure was added to explain how dominant structure of traffic networks changes with the moment order *q* (see Fig 2).

**Fig 2. Schematic representation of the filtering procedure for multifractal computation conducted by box-counting method with a regular grid of 16*16 boxes.** The probability distribution of traffic links is calculated over all boxes and then weighted by moment order *q*. Dark red boxes represent dominant structures with higher weighted probability, and dark blue boxes represent lightweight regions with lower weighted probability. (A) Partial traffic networks in Shenzhen. (B) When *q*=0, all non-empty boxes are equally weighted (gray). So the capacity dimension *D*_0_ is a measure of the space-filling capacity. (C) When *q*=1, it reflects the realistic density distribution of traffic networks. (D)-(E) For *q*>0, the boxes with relative high-density measures gradually gain more importance and their contribution to the entropy will dominate. (F) For *q*<0, the boxes with relatively low-density measures gradually gain more importance and their contribution to the entropy will dominate. In this way, with *q* changes from -∞ to ∞ step by step, the multifractal parameter spectrums correspond to urban fringes, main urban areas and central areas.

6. Similarly, estimating a power law with a simple OLS can be cumbersome. I advise to test their exponents with common packages as poweRlaw in R or similar in python. These take into accounts many errors that happen generally when using OLS directly.

**[Reply]** The algorithm based OLS has a significant advantage, that is, the algorithm is good for slope estimation (Chen, 2015). Where power law relation is concerned, the slope on a log-log plot represents fractal dimension or scaling exponent. Chen and his co-worker once employed OLS method and maximum likelihood method (MLM) to calculate the same allometric scaling exponents (Chen and Feng, 2017). The results show that if the power law relation is well developed, both the OLS and MLM will give similar calculation values. In contrast, if the power law is not well developed, the OLS can give approximate calculation values, while the MLM gives abnormal calculation values. This suggests that OLS can be used to make an approximate estimation of fractal dimension and scaling exponents, while the MLM can be utilized to identify fractals and nonfractals, power laws and fake power laws, and so on (Chen and Feng, 2017). In short, each method has its advantages and disadvantages. The algorithm of model parameter estimation can be selected according to the research objective and the characteristics of the research object

- Chen Y, Feng J (2017). A hierarchical allometric scaling analysis of Chinese cities: 1991-2014. *Discrete Dynamics in Nature and Society*, Volume 2017, Article ID 5243287, 15 pages
- Chen YG (2015). The distance-decay function of geographical gravity model: power law or exponential law? *Chaos, Solitons & Fractals*, 77: 174-189

7. The statistical analysis is not convincing to me. Showing an R-squared for a fixed intercept is not a good idea, as it loses its interpretation. No p-values are shown.

**[Reply]** In this revision, the goodness of fit (*R*^2^), the *p*-value, and the standard error of each parameter are given in Table 2, Table 3, and S3 Table.

The *R*-squared value can be transformed into the p-values easily. In fact, the fractal dimension calculation is based on one variable linear regression analysis. For one variable linear regression, the *R*^2, the *F* statistic, *t* statistic, and the corresponding *p*-value are equivalent to one another. What is more, the fractal dimension and the *R*^2 value can be associated with the standard error *δ*. The formulae are as follows

$F=t^{2}=\frac{vR^{2}}{1-R^{2}}$, (1)

$\delta=D\sqrt{\frac{1/R^{2}-1}{v}}$, (2)

where *v* denotes degree of freedom. If the intercept is free, the degree of freedom is *v*=*n*-2; If the intercept is fixed, the degree of freedom is *v*=*n*-1. Here *n* is the sample size, i.e., the datapoint. Then, using the function tdist, we can convert the *t* statistic into the corresponding p-value by means of MS Excel. The grammar is “=tdist(abs(*t* value), *v*, 2)”.

See the following example (Beijing, capacity dimension):

| Linear size of box, *r* | Number of nonempty box, *N*(*r*) | ln*r* | ln*N*(*r*) |
| --- | --- | --- | --- |
| 1 | 1 | 0 | 0 |
| 0.5 | 4 | -0.6931472 | 1.3862944 |
| 0.25 | 16 | -1.3862944 | 2.7725887 |
| 0.125 | 64 | -2.0794415 | 4.1588831 |
| 0.0625 | 256 | -2.7725887 | 5.5451774 |
| 0.03125 | 997 | -3.4657359 | 6.9047508 |
| 0.015625 | 3764 | -4.1588831 | 8.2332375 |
| 0.0078125 | 12041 | -4.8520303 | 9.3960728 |

**[Case 1]** Suppose that the intercept is free. In this case, the data point number is *n*=8, and the degree of freedom is *v*=*n*-2=6. The fractal dimension is *D*=1.95434, and the *R* squared is R^2=0.999499. The absolute value of *t* statistic is

$t=(\frac{vR^{2}}{1-R^{2}})^{0.5}=(\frac{6*0.999499}{1-0.999499})^{1/2}=109.355697$.

Then, input the formula “=tdist(109.355697,6,2)” into any cell of Excel, enter, we have the corresponding *p* value: 3.94E-11.

The standard error is

$$\delta=D\sqrt{\frac{1/R^{2}-1}{v}}=1.95434*(\frac{1/0.999499-1}{8-2})^{0.5}=0.017871$$

**[Case 2]** Suppose that the intercept is fixed to 0. In this case, the data point number is *n*=8, and the degree of freedom is *v*=*n*-1=7. The fractal dimension is *D*=1.97118, and the *R* squared is R^2=0.999821. The absolute value of *t* statistic is

$t=(\frac{vR^{2}}{1-R^{2}})^{0.5}=(\frac{7*0.999821}{1-0.999821})^{1/2}=197.615081$.

Then, input the formula “=tdist(197.615081,6,2)” into any cell of Excel, enter, we have the corresponding *p*-value: 2.24E-14.

The standard error is

$$\delta=D\sqrt{\frac{1/R^{2}-1}{v}}=1.97118*(\frac{1/0.999821-1}{8-1})^{0.5}=0.009975$$

See the response to the related comments of reviewer #3 and the following papers:

- Chen YG (2015). A new methodology of spatial cross-correlation analysis. *PLoS ONE*, 10(5): e0126158
- Chen YG (2018). Logistic models of fractal dimension growth of urban morphology. *Fractals*, 26(3): 1850033

8. The study is limited to Chinese cities, this is not a drawback, but should be mentioned as a limitation.

**[Reply]** Thank you for pointing out this limitation. We agree that our study is limited to Chinese cities, due to the lack of reliable street network data in other countries. This drawback has been added to the text in Line 412-413:

“Second, our data only covered Chinese cities. Further works might take traffic networks in other countries into account.”

9. This might be out of scope, but I advise the author to check the sensitivity to which the CCA is applied. An easy approach might be to just take the city center and consider a varying radius and see the influence thereof.

**[Reply]** Thanks for providing this insight. In this paper, we applied the CCA to identify the city boundary in a consistent way. The suggested experiment is interesting and would provide additional information about the spatial structures of urban traffic networks for an individual city across different spatial scales. Our concern is that, due to the various size of cities, it will be incomparable to use radius method to determine the studied urban area. Because some cities may cover more suburbs or rural areas under different radius. So it may be more suitable for vertical comparison within an individual city. Our future studies will try to further mine the hierarchical structure information by some “urban fingerprint” curves with multifractal parameters varying with radius.
