# Supplementary material for: Multifractal scaling analyses of urban street network structure: The cases of twelve megacities in China
Source: PLoS One. 2021 Feb 18;16(2):e0246925. doi: 10.1371/journal.pone.0246925 (PMC7891711; doi:10.1371/journal.pone.0246925)
Supplement: Attachment — Submitted filename: Response to Reviewers (R2)_0112.docx [file pone.0246925.s007.docx]

## Response to Reviewers (R2)

*Referring to the two rounds of comments of the reviewers, we have completely rewritten this article in recent days.*

### Reviewer #1:

**[Reviewer #1]**: I consider that the authors replied adequately to my comments. Even if I still have some reservations about the interpretation of the results given by the authors, I consider the paper at a good level for publication. Just a little remark:

Fig. 1 should be placed on the same page than its caption. Please, consider figure and its caption as a unique object.

**[Reply]** Thank you for your constructive comments. I'm sorry we can't handle all your comments. Sometimes, we can't consider all the problems clearly in the short term. Anyway, this time, referring to the two rounds of reviewers' comments, we have revised the full text. A new figure has been provided for the revised version (R2).

### Reviewer #3:

**[Reviewer #3]** Although authors have applied my minor comments and one of my major comments regarding the network links, yet in my opinion the paper is not sufficiently well-written to be useful for its target audience.

**[Reply]** Dear reviewer, you misunderstand the concept of conventional mathematical methods. You seem to be mad at the concept, but there is no derogatory meaning to this concept. Please see the following explanation.

**[Reviewer #3]** Authors have started their response by providing clarification on necessity and rigor of their study. The explanation (similar to the manuscript) contains too many grammatically incorrect sentences such as “Multifractal theory does be an excellent mathematical tool…” which makes the explanations hard to follow.

**[Reply]** Sorry for the clerical errors.

**[Reviewer #3]** In my opinion, authors misunderstood the comment about “over-complication of the problem at hand.” This means that simpler methods can deliver even a clearer and easier-to-interpret analysis of street networks.

**[Reply]** The research method of a problem does not lie in its simplicity or complexity, but in its effectiveness.

The greatest truths are the simplest, we know. Unfortunately, not all problems can be solved in a simple way.

For the simple aspects of traffic networks, we can make effective analyses by using simpler and clearer means. However, for the complex aspects of traffic networks, the simple approaches are limited in many cases.

**[Reviewer #3]** One of my concerns was ambiguity of arguments in this manuscript. Now in their response authors state that “Through the spectral curves, we can examine the different parts and different levels of a geographical system.” This is another example of ambiguity. What are “different levels of a geographical system”? There are numerous attributes that exist at different levels in a geographical system. If by this the authors mean, say, “different levels of granularity,” then they should write so in a clear and understandable manner. A publishable manuscript needs to contain minimum occurrence of such sentences.

**[Reply]** Dear reviewer, the following statement has no problem: “*Through the spectral curves, we can examine the different parts and different levels of a geographical system.*”

Let us explain it to you step by step:

(1) Geographical systems proved to be fractal-like systems, and a fractal system is a self-similar hierarchy with cascade structure.

(2) Any system has different parts. The basic principle of system theory is that “the whole is greater than the sum of parts” (please see general system theory).

(3) Any hierarchy has different levels. As indicated above, a fractal system is a self-similar hierarchy with cascade structure.

(4) A multifractal system is in fact a self-similar hierarchy with asymmetric cascade structure. Any multifractal system has different parts and levels.

The same sentence is clear for a person, but may be ambiguous for another person. Whether a sentence is clear to a person depends on his knowledge structure.

**[Reviewer #3]** Again, in comment 1 I quoted just a few sentences as examples which in my opinion do not explain the technical issues in a proper manner, e.g. “It is difficult to model and analyze complex networks by conventional mathematical methods.” Authors have responded by “Dear reviewer, all the following statements are easy to understand if you are familiar with multifractal theory.” This shows an issue with authors’ approach to technical writing; no matter the target audience, technical writing needs to be sound and clear. The problem with the quoted sentence has nothing to do with “familiarity with the topic,” i) you are making a wrong statement about conventional mathematical methods, which indeed are able to model and analyze complex networks, and ii) fractal analysis can also be deemed as a conventional mathematical method, but you are painting it differently as it is an advantage!

**[Reply]** Dear reviewer, mathematical methods fall into two categories: one is based on *characteristic scales* (characteristic length), and the other is based on *scaling*. For the purpose of naming and distinguishing, we call the first category “conventional mathematical methods”. A characteristic scale is always represented by a 1-dimensional measure such as radius, mean, eigenvalue, thus it is termed *characteristic length* in literature. What is called *conventional mathematical method* is the mathematical method based on *characteristic length*. The word “conventional” has no derogatory sense. We always make studies by using *conventional mathematical method*s.

The precondition of applying a mathematical method to modeling or analyzing a system is to find its characteristic scale (characteristic length). If so, we can use conventional mathematical methods (calculus, linear algebra, probability theory and statistics, etc.) to make analysis.

However, if you cannot find the characteristic scale (characteristic length) from a system, the conventional mathematical method will be ineffective. You can make analysis of the system without characteristic scale by using the conventional mathematical methods (there is no derogatory meaning ), but the explanation and prediction will be ineffective.

In this case, the characteristic scale (length) should be replaced by the scaling exponent such as fractal dimension.

| Type | Basis | Method | Scope of application | Note |
| --- | --- | --- | --- | --- |
| Conventional method (no derogatory sense) | Characteristic scale (Characteristic length) | Calculus, linear algebra, probability theory and statistics, etc. | Simple systems or simple aspects of complex systems | Simple systems have no complex aspect |
| Nonconventional method | Scaling (dilation symmetry) | Fractal geometry, complex network theory, wavelet analysis, renormalization group, etc. | Complex systems or complex aspects of complex systems | Complex systems may have simple aspects |

Cities and traffic networks are complex spatial systems. They have no characteristic scales in many aspects. You can use conventional mathematical methods to describe the simple aspects, but you cannot describe the complex aspects of these systems.

| Complex systems | Sign | Measure | Mathematical methods | Geographical model (example) |
| --- | --- | --- | --- | --- |
| Simple aspect | Characteristic length (typical scale) | Effective length, area, mean, standard deviation, eigenvalue, etc. | Calculus, linear algebra, probability theory and statistics, etc. | Clark model of population density |
| Complex aspect | Scaling (no characteristic length) | No effective length, area, mean, standard deviation, eigenvalue, etc. | Fractal geometry, complex network theory, wavelet analysis, renormalization group, etc. | Smeed model of urban traffic network |

**[Reviewer #3]** Authors refused to apply comment 3 and responded “Nonfractal systems have no fractal dimension.” This is just not true. A non-fractal system tends to have a flatter D_(Q) vs Q. This is one of the fundamental aspects of fractal analysis."

**[Reply]** Dear reviewer, you confuse monofractals (simple fractals) with nonfractals. A monofractal system has a flatter spectral line of *D*(*q*) vs *q*. This indicates that a simple fractal object bears a single fractal dimension value.

However, a nonfractal has topological dimension and Euclidean dimension, but has no fractal dimension.

Last time, you said, “There is a need for at least present a synthetic example of how a non-fractal network system would compare to those analysed in this paper. Perhaps by generating a network with insignificant fractality and then performing comparisons by means of D0, D1, and D2.”

The analysis methods for nonfractal systems are based on *characteristic scales*, fractal analyses are based on *scaling*. The technical routes of research are different.

We respect your comments very much, but we don’t know how to apply your comment 3 to this study. We are sorry.

### Reviewer #4:

**[Reviewer #4]**: I thank the authors for the revision of their manuscript. In the course of this evaluation, I have also read the reviews of the other reviewers. Interestingly, the third reviewer raises similar issues, especially about the over-complication. As a general comment, I still lack the connection to traffic itself. Table 1 is a good example of how the study still fails to demonstrate to show this connection.

**[Reply]** Multifractal modeling is a mathematical method. To our understanding, the basic property of a mathematical tool is its abstractness, and the powerful function of a mathematical tool lies in its abstractness.

Multifractal theory has been around for many years. However, its application in urban and traffic studies is limited. The problem lies in its abstractness.

We hope we can make multifractal analysis of traffic networks in a simple and visual way. Unfortunately, we do not know how to do for the time being. We hope to follow the direction proposed by you to do in the future.

How to apply multifractal approach to complex spatial systems is still in a course of exploring. We're not doing well enough at present, but we're trying to do well.

I hope you will support our developing research works.

**[Reviewer #4]** I believe that the authors have not addressed all of my comments appropriately. There are also multiple careless spelling errors in the revision letter. I still have open questions about the following responses.

**[Reply]** Many of your comments are interesting and constructive. Unfortunately, for the time being, we cannot address all of your comments. For example, the algorithm of maximum likelihood method.

**[Reviewer #4]** R1: The table does still not relate to traffic.

**[Reply]** Where the basic theory is concerned, we do not how to relate it to traffic according to your requirements. Where results and conclusions are concerned, our study is closely related to traffic networks.

**Table 5. The main results and inferences of calculations and analyses on multifractal traffic networks of 12 Chinese cities**

| Result and explanation | Inference |
| --- | --- |
| [Result] At the global level, the *D_q_* spectrums take on inverse S-shaped curves; at the local level, the *f*(*α*) spectrums take on unimodal curves. [Explanation] For monofractals, the *D_q_* spectrums become horizontal straight lines, and each *f*(*α*) spectrum condenses into a point. For nonfractals, no power laws, or *D*_0_=*D*_1_=*D*_2_=*d*. | The traffic networks of 12 cities in China are multifractal systems without exception. |
| [Result] When *q* →-∞, we have *D_q_*>>*d*=2; when *q* →∞, the scaling relations for local dimension are broken. [Explanation] When *q* →-∞, the multifractal spectrums reflect edge areas or network sparse regions; when *q* →∞, the multifractal spectrums mirror on central areas or network intensive regions. | The traffic networks of 12 cities in China take on disordered development in road sparse regions and periphery regions. |
| [Result] The scaling relation reflecting *α*(*q*) does not degenerate significantly with the change of *q*, but the scaling relation of *f*(*α*) partially degenerates when *q* value becomes too high or too low. [Explanation] The scaling relation degeneration means that the power-law distribution is broken, and the scattered points on the corresponding log-log plot do not take on a straight linear trend. | The traffic networks of 12 cities in China follow scaling law in different parts and levels, but the Scaling relation precedes fractal structure. |
| [Result] Local fractal dimension spectrums are of asymmetry. The left tails of *f*(*q*) curves are higher than right tails, while the left ends of *f*(*α*) curves is lower than the right ends. However, the tops of the *f*(*α*) curves slope to the left. [Explanation] Multifractal development falls into two categories: spatial concentration and spatial deconcentration. | The traffic networks of 12 cities in China take on spatial aggregation patterns, but include spatial diffusion trends. |

**[Reviewer #4]** R2: The aim is still unclear. What are the implications of the work, what do I learn from it? It still seems to be a research for the sake of research.

**[Reply]** The aim is to explore the general multifractal scaling properties in urban traffic networks by describing the scale-free aspects of network structure. We try to reveal the common regularities of traffic networks which bear no characteristic length. For complex systems, the problem is not single, nor can it be solved at one time.

Let us explain it step by step:

(1) Any scientific research includes two processes: one is *description* (for “how”), and the other is *understanding* (for “why”).

(2) Precise description is the precondition of deep understanding. However, precise description relies heavily on mathematics and measurements.

(3) For conventional mathematical method, effective description by modeling and measurements is to find *characteristic scales* (*characteristic lengths*). Unfortunately, complex spatial systems always have no characteristic length in many aspects. In this case, the characteristic scales (lengths) should be replaced by scaling exponents.

(4) Multifractal scaling can be used to describe complex systems with spatial heterogeneity such as cities and traffic networks.

We select a set of traffic networks of 12 cities in China. Obviously, we try to reveal the general multifractal scaling characteristics of urban street networks. Based on the common regularities, deep research on traffic networks can be made in the future.

The main results and conclusions are tabulated in Table 5. If you can reach these conclusions by other methods, we will withdraw the manuscript voluntarily.

**[Reviewer #4]** R6: I am somewhat doubtful about the OLS answer. I advise the authors to compare their results with the MLM approach. [Similarly, estimating a power law with a simple OLS can be cumbersome. I advise to test their exponents with common packages as power law in R or similar in python. These take into accounts many errors that happen generally when using OLS directly]

**[Reply]** The OLS is the best algorithm to estimate fractal dimension because it can give an effective slope of regression line based on power law. I know that many scholars are influenced by the following papers:

- Clauset A, Shalizi CR, Newman MEJ (2009). Power-law distributions in empirical data. *Siam Review*, 51(4): 661-703
- Newman MEJ (2005). Power laws, Pareto distributions and Zipf's law. *Contemporary Physics*, 46(5): 323-351

We respect your suggestion very much, but we are unable to change the algorithm for the time being. The reasons why we do not use the maximum likelihood method are as follows.

**First, the process of computing multifractal parameter spectrums is very complicated.** Especially, different algorithms may lead to very different results. If the algorithm is changed, the paper must be rewritten. If so, we have to write a new paper instead of revising a paper.

**Second, the premise of the validity of MLM is that the random variables satisfy the joint normal distribution.** For multifractals systems, we do not know if the linear scales and the corresponding measurements meet the joint Gaussian distribution.

**Third, if the scaling relation or power law is significant, both MLM and OLS give similar results to each other; in contrast, if the scaling relation or power law is not significant, the OLS will give approximate results, but the MLM will give abnormal results.** This suggests that the advantage of MLM is to distinguish real power laws from fake power laws. In terms of fractal dimension estimation, the maximum likelihood method is not superior to the least square method.

**Fourth, cities and traffic networks are evolutive fractal-like systems.** It is OLS rather than MLM that can be employed to examine the evolutive process of urban development.

**Fifth, the common packages based on R or python are in fact for bin data, rather than for the spatial data based on box-counting method.** In other words, they were designed for size distribution based on cross-sectional data rather than spatial distribution based on spatial data. To our understanding, the cross-sectional data extracted from a map is different from the spatial data used for multifractal parameter estimation.

Now, a paragraph has been added to the Discussion to discuss this question:

“Third, the algorithm of parameter estimation is limited to ordinary least squares (OLS) method. Perhaps the maximum likelihood method (MLM) can supplement some deficiency of the least squares calculation. MLM is regarded as a better approach to estimating power exponent values. However, the effectiveness of MLM depends on the joint normal distribution of random variables. Whether or not the linear size of boxes and corresponding measurements satisfy joint Gaussian distribution is unclear for the time being. The algorithm based on OLS has a significant advantage, that is, it is good for slope estimation. Where power law relation is concerned, the slope on a log-log plot represents fractal dimension or scaling exponent. In practice, both the OLS method and MLM can be employed to calculate scaling exponents. If the power law relation is well developed, the two algorithms will give similar calculation values. In contrast, if the power law is not well developed, the OLS can give approximate calculation values, while the MLM gives abnormal calculation values. This suggests that OLS can be used to make an approximate estimation of fractal dimension and scaling exponents, while the MLM can be utilized to distinguish power laws from fake power laws (Chen and Feng, 2017). In short, each method has its advantages and disadvantages. The algorithm of model parameter estimation can be selected according to the research objective and the characteristics of the research object. In this sense, MLM can be utilized to identify the disordered structure of multifractal traffic networks.”
