# Supplementary material for: Multifractal scaling analyses of urban street network structure: The cases of twelve megacities in China
Source: PLoS One. 2021 Feb 18;16(2):e0246925. doi: 10.1371/journal.pone.0246925 (PMC7891711; doi:10.1371/journal.pone.0246925)
Supplement: Attachment — Submitted filename: Response to Reviewers (R3)_2021-01-26.docx [file pone.0246925.s008.docx]

## Response to Reviewers (R3)

*The newly revised parts and added parts are written in red.*

### Reviewer #1:

**[Reviewer #1]**: I consider that the authors replied adequately to my comments. Even if I still have some reservations about the interpretation of the results given by the authors, I consider the paper at a good level for publication. Just a little remark:

Fig. 1 should be placed on the same page than its caption. Please, consider figure and its caption as a unique object.

**[Reply]** Thank you for your constructive comments. I'm sorry we can't handle all your comments. Sometimes, we can't consider all the problems clearly in the short term. Anyway, this time, referring to the two rounds of reviewers' comments, we have revised the full text. A new figure has been provided for the revised version (R2).

### Reviewer #4:

**[Reviewer #4]**: I thank the authors for the revision of their manuscript. In the course of this evaluation, I have also read the reviews of the other reviewers. Interestingly, the third reviewer raises similar issues, especially about the over-complication. As a general comment, I still lack the connection to traffic itself. Table 1 is a good example of how the study still fails to demonstrate to show this connection.

**[Reply]** A paragraph has been added to the Discussion part as below:

“Third, multifractal description os traffic network is not visual enough. In other words, the description and explanation of fractal parameters for urban street networks are too abstract to be understood by beginners. How to explain the results of multifractal description in simple terms is one of the directions in the future.”

**[Reviewer #4]** I believe that the authors have not addressed all of my comments appropriately. There are also multiple careless spelling errors in the revision letter. I still have open questions about the following responses.

**[Reply]** Many of your comments are interesting and constructive. We have made efforts to revise our manuscript according to your comments.

**[Reviewer #4]** R1: The table does still not relate to traffic.

**[Reply]** We have added a table (Table 5) to summarize our results and inferences. They are closely related to the spatial structure of traffic networks.

**Table 5. The main results and inferences of calculations and analyses on multifractal traffic networks of 12 Chinese cities**

| Result and explanation | Inference |
| --- | --- |
| [Result] At the global level, the *D_q_* spectrums take on inverse S-shaped curves; at the local level, the *f*(*α*) spectrums take on unimodal curves. [Explanation] For monofractals, the *D_q_* spectrums become horizontal straight lines, and each *f*(*α*) spectrum condenses into a point. For nonfractals, no power laws, or *D*_0_=*D*_1_=*D*_2_=*d*. | The traffic networks of 12 cities in China are multifractal systems without exception. |
| [Result] When *q* →-∞, we have *D_q_*>>*d*=2; when *q* →∞, the scaling relations for local dimension are broken. [Explanation] When *q* →-∞, the multifractal spectrums reflect edge areas or network sparse regions; when *q* →∞, the multifractal spectrums mirror on central areas or network intensive regions. | The traffic networks of 12 cities in China take on disordered development in road sparse regions and periphery regions. |
| [Result] The scaling relation reflecting *α*(*q*) does not degenerate significantly with the change of *q*, but the scaling relation of *f*(*α*) partially degenerates when *q* value becomes too high or too low. [Explanation] The scaling relation degeneration means that the power-law distribution is broken, and the scattered points on the corresponding log-log plot do not take on a straight linear trend. | The traffic networks of 12 cities in China follow scaling law in different parts and levels, but the scaling relation precedes fractal structure. |
| [Result] Local fractal dimension spectrums are of asymmetry. The left tails of *f*(*q*) curves are higher than right tails, while the left ends of *f*(*α*) curves is lower than the right ends. However, the tops of the *f*(*α*) curves slope to the left. [Explanation] Multifractal development falls into two categories: spatial concentration and spatial deconcentration. | The traffic networks of 12 cities in China take on spatial aggregation patterns, but include spatial diffusion trends. |

**[Reviewer #4]** R2: The aim is still unclear. What are the implications of the work, what do I learn from it? It still seems to be a research for the sake of research.

**[Reply]** The aim is to explore the general multifractal scaling properties in urban traffic networks by describing the scale-free aspects of network structure. We try to reveal the common regularities of traffic networks which bear no characteristic length. For complex systems, the problem is not single, nor can it be solved at one time.

Let us explain it step by step:

(1) Any scientific research includes two processes: one is *description* (for “how”), and the other is *understanding* (for “why”).

(2) Precise description is the precondition of deep understanding. However, precise description relies heavily on mathematics and measurements.

(3) For conventional mathematical method, effective description by modeling and measurements is to find *characteristic scales* (*characteristic lengths*). Unfortunately, complex spatial systems always have no characteristic length in many aspects. In this case, the characteristic scales (lengths) should be replaced by scaling exponents.

(4) Complexity theory inspires a new way of looking at traffic networks from the bottom up and self-organization. Multifractal scaling can be used to describe complex systems with spatial heterogeneity such as cities and traffic networks. These are regarded as important for understanding the underlying mechanism and characteristics of traffic networks and are of great significance in the rational spatial optimization of urban traffic systems layout.

We select a set of traffic networks of 12 cities in China. Obviously, we try to reveal the general multifractal scaling characteristics of urban street networks. Based on the common regularities, deep research on traffic networks can be made in the future.

We have reorganized the Introduction section to better clarify the objectives of the paper:

“Complex system evolution is mainly based on multifractal scaling processes rather than monofractal scaling process. Now, there is no doubt that the traffic networks have statistical self-similar structure. However, it is not very clear whether the spatial structure of traffic networks in China are of monofractal structure or multifractal structure. Before deeply studying the complexity of traffic networks, we must first find out whether the traffic networks generally have multifractal structure. If so, what are the common characteristics of different traffic networks? Only by properly describing the general multiscaling characteristics of traffic networks, can we expect to further research the evolution mechanism behind them. Therefore, this paper is devoted to exploring the general regularities of multifractal structure of urban traffic networks represented by street links. The aim is at providing some empirical foundations for further research on internal mechanisms and general principles of complex spatial networks.”

The main results and conclusions are tabulated in Table 5.

First of all, through several cases, we found that similar to the spatial distribution of urban population and urban land, the spatial structure of urban street network also exhibits universal multifractal characteristics. The urban traffic network system can be regarded as the systematic combination of local subsystems, which form a cascade structure through continuous self-replication.

Second, from the local perspective, we revealed the spatial characteristics of different local subsystems of urban street network. On the one hand, the degradation of fractal structure was observed in the central areas. This implies that the spatial pattern of urban street network in the central area tends to be relatively compact and saturated. On the other hand, the urban street network presents disorderly and nonsystematic spread in marginal areas. This implies that the hierarchical structure of traffic networks has not been well developed in suburban areas.

Lastly, from the global perspective, the fractal growth of urban sreet networks presents a spatial concentration pattern. Some central areas occupy a large number of traffic lines. The spatial pattern of urban street network is coordinated with its connectivity structure, and forms a hierarchical cascade structure. However, it also shows a potential diffusion tendency in space. This may be related to the urban sprawl and expansion, or the spatial development of traffic networks is in a certain transformation period.

The implication of this work lies in two aspects. First of all, through case studies, an in-depth understanding of the multifractal characteristics of traffic network is provided, which is helpful for further research on underlying mechanisms and general principles of complex spatial networks. Second, the multifractal patterns of traffic networks revealed by this study can provide a planning reference for the effective spatial organization of urban traffic network system. The development of urban street network is not only an organic growth process as a whole system, but also an interactive process of several subsystems. In the long term, to improve traffic efficiency, we should optimize traffic organization and management in central areas. And special efforts should be made to improve the unified planning of suburban street network and strengthen the systematic construction.

**[Reviewer #4]** R6: I am somewhat doubtful about the OLS answer. I advise the authors to compare their results with the MLM approach. [Similarly, estimating a power law with a simple OLS can be cumbersome. I advise to test their exponents with common packages as power law in R or similar in python. These take into accounts many errors that happen generally when using OLS directly]

**[Reply]** The OLS is the best algorithm to estimate fractal dimension because it can give an effective slope of regression line based on power law. I know that many scholars are influenced by the following papers:

- Clauset A, Shalizi CR, Newman MEJ (2009). Power-law distributions in empirical data. *Siam Review*, 51(4): 661-703
- Newman MEJ (2005). Power laws, Pareto distributions and Zipf's law. *Contemporary Physics*, 46(5): 323-351

We respect your suggestion very much, but we are unable to change the algorithm for the time being. The reasons why we do not use the maximum likelihood method are as follows.

**First, the process of computing multifractal parameter spectrums is very complicated.** Especially, different algorithms may lead to very different results. If the algorithm is changed, the paper must be rewritten. If so, we have to write a new paper instead of revising a paper.

**Second, the premise of the validity of MLM is that the random variables satisfy the joint normal distribution.** For multifractals systems, we do not know if the linear scales and the corresponding measurements meet the joint Gaussian distribution.

**Third, if the scaling relation or power law is significant, both MLM and OLS give similar results to each other; in contrast, if the scaling relation or power law is not significant, the OLS will give approximate results, but the MLM will give abnormal results.** This suggests that the advantage of MLM is to distinguish real power laws from fake power laws. In terms of fractal dimension estimation, the maximum likelihood method is not superior to the least square method.

**Fourth, cities and traffic networks are evolutive fractal-like systems.** It is OLS rather than MLM that can be employed to examine the evolutive process of urban development.

**Fifth, the common packages based on R or python are in fact for bin data, rather than for the spatial data based on box-counting method.** In other words, they were designed for size distribution based on cross-sectional data rather than spatial distribution based on spatial data. To our understanding, the cross-sectional data extracted from a map is different from the spatial data used for multifractal parameter estimation.

As suggested, we have tried the regression estimation by using MLM method in R, and calculated the multifractal parameters. In our cases, the results show that there is no significant difference between the OLS method and MLM method, taking Beijing as an example (Table A1~A3). The generalized correlation dimensions *D*(*q*) (Table A1) and the singularity exponents *α*(*q*) (Table A2) are all significant at the level 1%. While for the local dimension *f* (*q*) (Table A3), when the value of moment order *q* increases to a certain range (*q* = 20~40), the significance level decreases quickly.

The relationships between the multifractal parameters of Beijing’s street network based on OLS and those based on MLM are displayed in Figures A-C, which show that there is no significant difference between the OLS-based calculated results and the MLM-based calculated results.

MLM

| OLS | D(q) |
| --- | --- |
| 3.14202 | 3.14205 |
| 3.14041 | 3.14041 |
| 3.13872 | 3.13874 |
| 3.13694 | 3.13694 |
| 3.13506 | 3.13507 |
| 3.13308 | 3.13307 |
| 3.13099 | 3.13098 |
| 3.12877 | 3.1288 |
| 3.12641 | 3.1264 |
| 3.1239 | 3.12386 |
| 3.12123 | 3.12124 |
| 3.11838 | 3.11838 |
| 3.11534 | 3.11535 |
| 3.11207 | 3.11208 |
| 3.10856 | 3.10856 |
| 3.10477 | 3.10476 |
| 3.10068 | 3.10068 |
| 3.09624 | 3.09624 |
| 3.09141 | 3.09141 |
| 3.08614 | 3.08611 |
| 3.08035 | 3.08035 |
| 3.07398 | 3.07432 |
| 3.06693 | 3.06691 |
| 3.05909 | 3.05907 |
| 3.0503 | 3.0503 |
| 3.0404 | 3.04038 |
| 3.02916 | 3.02916 |
| 3.01628 | 3.0163 |
| 3.00138 | 3.00138 |
| 2.98396 | 2.98396 |
| 2.96332 | 2.96333 |
| 2.93849 | 2.93848 |
| 2.90806 | 2.90806 |
| 2.86994 | 2.86994 |
| 2.82088 | 2.82088 |
| 2.75556 | 2.75556 |
| 2.66472 | 2.66471 |
| 2.53107 | 2.53108 |
| 2.32187 | 2.32187 |
| 2.03744 | 2.03744 |
| 2.01501 | 2.01501 |
| 1.99542 | 1.99542 |
| 1.97859 | 1.97859 |
| 1.96425 | 1.96425 |
| 1.95202 | 1.95202 |
| 1.94151 | 1.94152 |
| 1.93237 | 1.93237 |
| 1.92431 | 1.92432 |
| 1.91709 | 1.91709 |
| 1.91055 | 1.91055 |
| 1.90455 | 1.90455 |
| 1.89899 | 1.89899 |
| 1.89379 | 1.89379 |
| 1.88891 | 1.88891 |
| 1.88429 | 1.88429 |
| 1.8799 | 1.8799 |
| 1.87571 | 1.87571 |
| 1.87171 | 1.87171 |
| 1.86788 | 1.86788 |
| 1.8642 | 1.8642 |
| 1.83388 | 1.83388 |
| 1.81176 | 1.81176 |
| 1.79471 | 1.79471 |
| 1.78089 | 1.78089 |
| 1.76921 | 1.76921 |
| 1.75897 | 1.75897 |
| 1.74974 | 1.74974 |
| 1.74121 | 1.74121 |
| 1.73321 | 1.73321 |
| 1.72564 | 1.72564 |
| 1.71847 | 1.71847 |
| 1.71172 | 1.71172 |
| 1.70539 | 1.7054 |
| 1.69951 | 1.69951 |
| 1.69406 | 1.69406 |
| 1.68904 | 1.68904 |
| 1.68442 | 1.68442 |
| 1.68017 | 1.68017 |
| 1.67626 | 1.67626 |
| 1.67267 | 1.67267 |
| 1.66936 | 1.66936 |
| 1.66631 | 1.66631 |
| 1.66349 | 1.66349 |
| 1.66088 | 1.66088 |
| 1.65846 | 1.65846 |
| 1.6562 | 1.6562 |
| 1.6541 | 1.6541 |
| 1.65214 | 1.65214 |
| 1.65031 | 1.65031 |
| 1.64859 | 1.64859 |
| 1.64698 | 1.64698 |
| 1.64546 | 1.64546 |
| 1.64403 | 1.64403 |
| 1.64269 | 1.64269 |
| 1.64141 | 1.64141 |
| 1.64021 | 1.64021 |
| 1.63907 | 1.63907 |
| 1.63798 | 1.63798 |
| 1.63695 | 1.63695 |

Figure A The generalized correlation dimension values of Beijing’s street network based on OLS and MLM

MLM

| OLS | α(q) |
| --- | --- |
| 3.20628 | 3.2372 |
| 3.2063 | 3.23716 |
| 3.20634 | 3.23714 |
| 3.20637 | 3.23711 |
| 3.2064 | 3.23707 |
| 3.20644 | 3.23705 |
| 3.20648 | 3.23702 |
| 3.20652 | 3.237 |
| 3.20657 | 3.23699 |
| 3.20662 | 3.23697 |
| 3.20667 | 3.23697 |
| 3.20673 | 3.23698 |
| 3.2068 | 3.23698 |
| 3.20687 | 3.237 |
| 3.20695 | 3.23704 |
| 3.20704 | 3.23708 |
| 3.20714 | 3.23715 |
| 3.20725 | 3.23724 |
| 3.20737 | 3.23735 |
| 3.20751 | 3.23749 |
| 3.20767 | 3.23767 |
| 3.20785 | 3.23786 |
| 3.20805 | 3.23811 |
| 3.20829 | 3.2384 |
| 3.20856 | 3.23878 |
| 3.20888 | 3.23922 |
| 3.20924 | 3.2398 |
| 3.20966 | 3.24057 |
| 3.21014 | 3.24162 |
| 3.2107 | 3.24307 |
| 3.21133 | 3.24511 |
| 3.21202 | 3.24778 |
| 3.21271 | 3.25066 |
| 3.21326 | 3.25159 |
| 3.21331 | 3.37438 |
| 3.21194 | 3.24952 |
| 3.20656 | 3.06535 |
| 3.18823 | 3.18823 |
| 3.10629 | 3.1063 |
| 2.51358 | 2.51358 |
| 2.41443 | 2.41443 |
| 2.32268 | 2.32268 |
| 2.24255 | 2.24255 |
| 2.17571 | 2.17571 |
| 2.12158 | 2.12158 |
| 2.0783 | 2.0783 |
| 2.04365 | 2.04365 |
| 2.01559 | 2.01559 |
| 1.99249 | 1.99249 |
| 1.97308 | 1.97309 |
| 1.95645 | 1.95645 |
| 1.94192 | 1.94192 |
| 1.92902 | 1.92902 |
| 1.91739 | 1.91739 |
| 1.90679 | 1.90679 |
| 1.89703 | 1.89703 |
| 1.88798 | 1.88798 |
| 1.87954 | 1.87954 |
| 1.87163 | 1.87163 |
| 1.8642 | 1.8642 |
| 1.80834 | 1.80834 |
| 1.77337 | 1.77337 |
| 1.74909 | 1.74909 |
| 1.73045 | 1.73045 |
| 1.71486 | 1.71486 |
| 1.70092 | 1.70092 |
| 1.68784 | 1.68783 |
| 1.67527 | 1.67527 |
| 1.66322 | 1.66322 |
| 1.65198 | 1.65198 |
| 1.64189 | 1.64189 |
| 1.63321 | 1.63321 |
| 1.62601 | 1.62601 |
| 1.62019 | 1.62019 |
| 1.61557 | 1.61557 |
| 1.61195 | 1.61195 |
| 1.60912 | 1.60912 |
| 1.6069 | 1.6069 |
| 1.60516 | 1.60516 |
| 1.60378 | 1.60378 |
| 1.60269 | 1.60269 |
| 1.60181 | 1.60181 |
| 1.60111 | 1.60111 |
| 1.60053 | 1.60053 |
| 1.60006 | 1.60006 |
| 1.59968 | 1.59968 |
| 1.59936 | 1.59936 |
| 1.59909 | 1.59909 |
| 1.59886 | 1.59887 |
| 1.59868 | 1.59868 |
| 1.59851 | 1.59851 |
| 1.59838 | 1.59838 |
| 1.59826 | 1.59826 |
| 1.59816 | 1.59816 |
| 1.59807 | 1.59807 |
| 1.598 | 1.598 |
| 1.59793 | 1.59793 |
| 1.59787 | 1.59787 |
| 1.59782 | 1.59782 |

Figure B The singularity exponent values of Beijing’s street network based on OLS and MLM

f(q)

MLM

| OLS | f(q) |
| --- | --- |
| 0.571728 | 0.571789 |
| 0.570596 | 0.570597 |
| 0.569423 | 0.569423 |
| 0.568204 | 0.568208 |
| 0.566936 | 0.566936 |
| 0.565616 | 0.565615 |
| 0.564236 | 0.564234 |
| 0.562793 | 0.562793 |
| 0.561281 | 0.56128 |
| 0.559692 | 0.55969 |
| 0.558018 | 0.558016 |
| 0.55625 | 0.556249 |
| 0.554379 | 0.554378 |
| 0.552394 | 0.552392 |
| 0.550281 | 0.550281 |
| 0.548026 | 0.548026 |
| 0.545614 | 0.545614 |
| 0.543027 | 0.543026 |
| 0.540245 | 0.540244 |
| 0.537246 | 0.537246 |
| 0.534007 | 0.534003 |
| 0.530502 | 0.530498 |
| 0.526703 | 0.5267 |
| 0.522581 | 0.522579 |
| 0.518107 | 0.518171 |
| 0.513253 | 0.513211 |
| 0.507998 | 0.507997 |
| 0.502331 | 0.502332 |
| 0.496264 | 0.496158 |
| 0.489854 | 0.489795 |
| 0.483243 | 0.483215 |
| 0.476722 | 0.476821 |
| 0.470858 | 0.470858 |
| 0.466719 | 0.466766 |
| 0.466329 | 0.466342 |
| 0.473662 | 0.473658 |
| 0.497317 | 0.497323 |
| 0.559625 | 0.559618 |
| 0.75302 | 0.753013 |
| 1.56131 | 1.56131 |
| 1.65554 | 1.65554 |
| 1.73361 | 1.73361 |
| 1.79381 | 1.79381 |
| 1.83737 | 1.83737 |
| 1.86724 | 1.86724 |
| 1.8868 | 1.8868 |
| 1.89899 | 1.89897 |
| 1.90605 | 1.90605 |
| 1.90955 | 1.90955 |
| 1.91055 | 1.91055 |
| 1.90974 | 1.90974 |
| 1.90757 | 1.90757 |
| 1.90436 | 1.90436 |
| 1.9003 | 1.9003 |
| 1.89554 | 1.89554 |
| 1.89018 | 1.89018 |
| 1.8843 | 1.8843 |
| 1.87798 | 1.87798 |
| 1.87126 | 1.87126 |
| 1.8642 | 1.8642 |
| 1.7828 | 1.7828 |
| 1.69659 | 1.69659 |
| 1.61225 | 1.61225 |
| 1.52869 | 1.52869 |
| 1.44314 | 1.44313 |
| 1.35262 | 1.35262 |
| 1.25456 | 1.25456 |
| 1.14775 | 1.14775 |
| 1.03337 | 1.03337 |
| 0.915405 | 0.915385 |
| 0.799501 | 0.7995 |
| 0.691107 | 0.691108 |
| 0.593969 | 0.593967 |
| 0.509717 | 0.509715 |
| 0.438281 | 0.438281 |
| 0.378564 | 0.378562 |
| 0.329022 | 0.329022 |
| 0.288032 | 0.288031 |
| 0.254097 | 0.254097 |
| 0.225922 | 0.225922 |
| 0.202432 | 0.202437 |
| 0.18275 | 0.18275 |
| 0.16617 | 0.16617 |
| 0.152128 | 0.152128 |
| 0.140172 | 0.140172 |
| 0.129938 | 0.129938 |
| 0.121135 | 0.12114 |
| 0.113525 | 0.113525 |
| 0.106917 | 0.106918 |
| 0.101152 | 0.101168 |
| 0.0961029 | 0.0961048 |
| 0.0916611 | 0.0916538 |
| 0.087738 | 0.0877396 |
| 0.0842592 | 0.0842591 |
| 0.0811625 | 0.0811626 |
| 0.0783951 | 0.0783959 |
| 0.0759125 | 0.0759152 |
| 0.0736767 | 0.0736768 |
| 0.0716555 | 0.0716555 |

Figure C The local fractal dimension values of Beijing’s street network based on OLS and MLM

Table A1 The generalized correlation dimension *D*(*q*) estimated by the OLS and MLM regression.

|  | OLS | | | MLM | | |
| --- | --- | --- | --- | --- | --- | --- |
| *q* | *D*(*q*) | Std Error | *p*-value | *D*(*q*) | Std Error | *p*-value |
| -40 | 3.142018^***^ | 0.2602784 | 2.05E-06 | 3.142053^***^ | 0.2328026 | 1.64E-41 |
| -35 | 3.133083^***^ | 0.2586764 | 2.00E-06 | 3.133073^***^ | 0.2313681 | 8.89E-42 |
| -30 | 3.121233^***^ | 0.2565522 | 1.93E-06 | 3.121236^***^ | 0.2294673 | 3.89E-42 |
| -25 | 3.10477^***^ | 0.2536017 | 1.84E-06 | 3.10476^***^ | 0.2268285 | 1.20E-42 |
| -20 | 3.080353^***^ | 0.2492289 | 1.71E-06 | 3.080353^***^ | 0.2229172 | 1.97E-43 |
| -15 | 3.040399^***^ | 0.2420871 | 1.51E-06 | 3.040379^***^ | 0.2165296 | 8.70E-45 |
| -10 | 2.963324^***^ | 0.2283691 | 1.18E-06 | 2.963325^***^ | 0.2042597 | 1.08E-47 |
| -5 | 2.755561^***^ | 0.1917371 | 5.37E-07 | 2.755561^***^ | 0.1714952 | 4.29E-58 |
| -4 | 2.664715^***^ | 0.1758088 | 3.55E-07 | 2.664714^***^ | 0.1572488 | 2.07E-64 |
| -3 | 2.531075^***^ | 0.1523623 | 1.74E-07 | 2.531076^***^ | 0.1362768 | 5.31E-77 |
| -2 | 2.321867^***^ | 0.1148617 | 3.75E-08 | 2.321867^***^ | 0.1027355 | 4.29E-113 |
| -1 | 2.037444^***^ | 0.05365968 | 2.54E-10 | 2.037444^***^ | 0.04799512 | 0 |
| 0 | 1.91055^***^ | 0.02506826 | 9.79E-13 | 1.910551^***^ | 0.02242252 | 0 |
| 1 | 1.864196^***^ | 0.01550655 | 2.56E-14 | 1.864195^***^ | 0.0138709 | 0 |
| 2 | 1.833876^***^ | 0.01261545 | 5.61E-15 | 1.833876^***^ | 0.01128546 | 0 |
| 3 | 1.81176^***^ | 0.013853 | 1.31E-14 | 1.811761^***^ | 0.01239186 | 0 |
| 4 | 1.794708^***^ | 0.01589033 | 4.22E-14 | 1.794708^***^ | 0.01421419 | 0 |
| 5 | 1.78089^***^ | 0.01762346 | 1.03E-13 | 1.78089^***^ | 0.01576323 | 0 |
| 10 | 1.733207^***^ | 0.02191277 | 7.28E-13 | 1.733207^***^ | 0.01960044 | 0 |
| 15 | 1.699508^***^ | 0.02396959 | 1.74E-12 | 1.699509^***^ | 0.0214396 | 0 |
| 20 | 1.676264^***^ | 0.02537022 | 3.06E-12 | 1.676264^***^ | 0.02269275 | 0 |
| 25 | 1.660882^***^ | 0.02616795 | 4.22E-12 | 1.660882^***^ | 0.02340623 | 0 |
| 30 | 1.650311^***^ | 0.02661869 | 5.09E-12 | 1.650311^***^ | 0.02380928 | 0 |
| 35 | 1.642687^***^ | 0.02689075 | 5.73E-12 | 1.642687^***^ | 0.02405268 | 0 |
| 40 | 1.636954^***^ | 0.02706629 | 6.21E-12 | 1.636955^***^ | 0.02420953 | 0 |

**Note:** ^***^ significant at 1%; ^**^ significant at 5%; ^*^ significant at 10%.

Table A2 The singularity exponent *α*(*q*) estimated by the OLS and MLM regression.

|  | OLS | | | MLM | | |
| --- | --- | --- | --- | --- | --- | --- |
| *q* | *α*(*q*) | Std Error | *p*-value | *α*(*q*) | Std Error | *p*-value |
| -40 | 3.206275^***^ | 0.271812 | 2.44E-06 | 3.237196^***^ | 0.7309329 | 9.47E-06 |
| -35 | 3.206439^***^ | 0.271845 | 2.44E-06 | 3.237047^***^ | 0.6903155 | 2.74E-06 |
| -30 | 3.206674^***^ | 0.2718905 | 2.45E-06 | 3.236966^***^ | 0.6453887 | 5.29E-07 |
| -25 | 3.20704^***^ | 0.2719564 | 2.45E-06 | 3.237081^***^ | 0.5979997 | 6.19E-08 |
| -20 | 3.20767^***^ | 0.2720581 | 2.45E-06 | 3.237666^***^ | 0.5513865 | 4.31E-09 |
| -15 | 3.208876^***^ | 0.2722268 | 2.46E-06 | 3.239216^***^ | 0.5019159 | 1.09E-10 |
| -10 | 3.211332^***^ | 0.2725007 | 2.46E-06 | 3.245112^***^ | 0.6032696 | 7.48E-08 |
| -5 | 3.211941^***^ | 0.2721641 | 2.43E-06 | 3.249515^***^ | 0.4386256 | 1.28E-13 |
| -4 | 3.206565^***^ | 0.2712065 | 2.40E-06 | 3.06535^***^ | 0.2392653 | 1.41E-37 |
| -3 | 3.188225^***^ | 0.2687683 | 2.34E-06 | 3.188225^***^ | 0.2403938 | 3.82E-40 |
| -2 | 3.10629^***^ | 0.2628133 | 2.41E-06 | 3.106296^***^ | 0.2350711 | 7.26E-40 |
| -1 | 2.513577^***^ | 0.1841212 | 7.98E-07 | 2.513577^***^ | 0.1646832 | 1.3468E-52 |
| 0 | 1.973085^***^ | 0.03868061 | 2.42E-11 | 1.973086^***^ | 0.03459741 | 0 |
| 1 | 1.864196^***^ | 0.01550655 | 2.56E-14 | 1.864195^***^ | 0.0138709 | 0 |
| 2 | 1.808339^***^ | 0.01382208 | 1.30E-14 | 1.808339^***^ | 0.01236451 | 0 |
| 3 | 1.773369^***^ | 0.01903777 | 1.97E-13 | 1.77337^***^ | 0.0170291 | 0 |
| 4 | 1.749094^***^ | 0.02226606 | 7.69E-13 | 1.749094^***^ | 0.01991642 | 0 |
| 5 | 1.730449^***^ | 0.02392079 | 1.49E-12 | 1.730449^***^ | 0.02139562 | 0 |
| 10 | 1.663223^***^ | 0.02714116 | 5.59E-12 | 1.663224^***^ | 0.02427658 | 0 |
| 15 | 1.620189^***^ | 0.03029621 | 1.66E-11 | 1.620189^***^ | 0.02709848 | 0 |
| 20 | 1.605156^***^ | 0.03014006 | 1.71E-11 | 1.605155^***^ | 0.02695882 | 0 |
| 25 | 1.600532^***^ | 0.02934406 | 1.42E-11 | 1.600532^***^ | 0.0262469 | 0 |
| 30 | 1.598864^***^ | 0.02879623 | 1.23E-11 | 1.598866^***^ | 0.0257567 | 0 |
| 35 | 1.59816^***^ | 0.02846292 | 1.12E-11 | 1.598161^***^ | 0.02545877 | 0 |
| 40 | 1.597822^***^ | 0.02825142 | 1.06E-11 | 1.597821^***^ | 0.02526952 | 0 |

**Note:** ^***^ significant at 1%; ^**^ significant at 5%; ^*^ significant at 10%.

Table A3 The local fractal dimension *f*(*q*) estimated by the OLS and MLM regression.

|  | OLS | | | MLM | | |
| --- | --- | --- | --- | --- | --- | --- |
| *q* | *f*(*q*) | Std Error | *p*-value | *f*(*q*) | Std Error | *p*-value |
| -40 | 0.5717276^**^ | 0.2281531 | 0.0366 | 0.5717889^***^ | 0.2040721 | 0.0051 |
| -35 | 0.5656155^**^ | 0.2283853 | 0.0383 | 0.5656154^***^ | 0.2042741 | 0.0056 |
| -30 | 0.5580177^**^ | 0.2284576 | 0.0404 | 0.5580162^***^ | 0.2043399 | 0.0063 |
| -25 | 0.5480262^**^ | 0.2282809 | 0.0431 | 0.5480262^***^ | 0.2041807 | 0.0073 |
| -20 | 0.534007^**^ | 0.2278299 | 0.0471 | 0.5340028^***^ | 0.2037798 | 0.0088 |
| -15 | 0.5132534^*^ | 0.2273251 | 0.0539 | 0.5132112^**^ | 0.2033215 | 0.0116 |
| -10 | 0.4832435^*^ | 0.2269716 | 0.0659 | 0.4832154^**^ | 0.2030236 | 0.0173 |
| -5 | 0.4736615^*^ | 0.2193616 | 0.0629 | 0.473658^**^ | 0.1961772 | 0.0158 |
| -4 | 0.4973168^**^ | 0.213243 | 0.0480 | 0.4973229^***^ | 0.1907303 | 0.0091 |
| -3 | 0.5596246^**^ | 0.2039621 | 0.0253 | 0.5596183^***^ | 0.1824292 | 0.0022 |
| -2 | 0.7530205^***^ | 0.1901174 | 0.0042 | 0.7530134^***^ | 0.170045 | 0.0000 |
| -1 | 1.561311^***^ | 0.09171724 | 0.0000 | 1.561311^***^ | 0.08203468 | 0.0000 |
| 0 | 1.91055^***^ | 0.02506826 | 0.0000 | 1.910551^***^ | 0.02242201 | 0.0000 |
| 1 | 1.864196^***^ | 0.01550655 | 0.0000 | 1.864196^***^ | 0.0138706 | 0.0000 |
| 2 | 1.782801^***^ | 0.01712249 | 0.0000 | 1.782802^***^ | 0.01531619 | 0.0000 |
| 3 | 1.696587^***^ | 0.03265092 | 0.0000 | 1.696587^***^ | 0.02920405 | 0.0000 |
| 4 | 1.61225^***^ | 0.04387933 | 0.0000 | 1.61225^***^ | 0.03924738 | 0.0000 |
| 5 | 1.528687^***^ | 0.05062936 | 0.0000 | 1.528688^***^ | 0.0452844 | 0.0000 |
| 10 | 1.033373^***^ | 0.09241786 | 0.0000 | 1.033373^***^ | 0.08266121 | 0.0000 |
| 15 | 0.5097169^**^ | 0.1686941 | 0.0165 | 0.5097155^***^ | 0.1508846 | 0.0007 |
| 20 | 0.2540965 | 0.1705367 | 0.1746 | 0.2540967^*^ | 0.152533 | 0.0957 |
| 25 | 0.1521284 | 0.1500485 | 0.3403 | 0.1521284 | 0.1342075 | 0.2570 |
| 30 | 0.1069167 | 0.1316629 | 0.4403 | 0.1069178 | 0.117763 | 0.3639 |
| 35 | 0.08425925 | 0.1179172 | 0.4952 | 0.08425911 | 0.1054685 | 0.4243 |
| 40 | 0.07165547 | 0.1076581 | 0.5244 | 0.07165546 | 0.09629251 | 0.4568 |

**Note:** ^***^ significant at 1%; ^**^ significant at 5%; ^*^ significant at 10%.

Now, a paragraph has been added to the Discussion to discuss this question:

“Fourth, the algorithm of parameter estimation was limited to the ordinary least squares (OLS) method. The alternative algorithm of OLS is the maximum likelihood method (MLM). MLM is regarded as a better approach to estimating power exponent values. However, the effectiveness of MLM depends on the joint normal distribution of random variables. Whether or not the linear size of boxes and corresponding measurements satisfy joint Gaussian distribution is unclear for the time being. The algorithm based on OLS has a significant advantage, that is, it is good for slope estimation. Where power law relation is concerned, the slope on a log-log plot represents fractal dimension or scaling exponent. In practice, both the OLS method and MLM can be employed to calculate scaling exponents. If the power law relation is well developed, the two algorithms will give similar calculation values. In contrast, if the power law is not well developed, the OLS can give approximate calculation values, while the MLM gives abnormal calculation values. This suggests that OLS can be used to make an approximate estimation of fractal dimension and scaling exponents, while the MLM can be utilized to distinguish power laws from fake power laws [72]. In short, each method has its advantages and disadvantages. The algorithm of model parameter estimation can be selected according to the research objective and the characteristics of the research object. In this sense, MLM can be utilized to identify the disordered structure of multifractal traffic networks. We have tested the regression estimation by using MLM in R, and calculated the multifractal parameters. In our cases, the two algorithms lead to the similar calculated results. For example, for Beijing, the values of generalized correlation dimensions Dq and local dimension f(q) based on OLS are highly consistent with the those based on MLM. As for the singularity exponents α(q), there are two outliers when q=-4 and -6. But the two outliers have no significant influence to the analytical conclusions (S4 Table). In short, the results show that there is no significant differences between the OLS-based multifractal spectrums and those based on MLM. The consistency between MLM-based results and the OLS-based results suggest that the spatial structure of urban street networks well follows power laws.”
